# Influences on tidal channel and aquaculture shrimp pond water chemical composition in Southwest Bangladesh

**DOI:** 10.1186/s12932-021-00074-2

**Published:** 2021-05-28

**Authors:** Matthew Dietrich, John C. Ayers

**Affiliations:** Department of Earth and Environmental Sciences, Vanderbilt University, 5726 Stevenson Center, 7th floor, Nashville, TN 37240 USA

**Keywords:** Trace elements, Arsenic, Selenium, Surface water chemistry, Aquaculture

## Abstract

Detailed geochemical studies of both major and minor elements in Bangladesh surface waters are sparse, particularly in shrimp aquaculture pond environments. Therefore, water samples from shrimp aquaculture ponds and tidal channels were collected in high precipitation (July) and low precipitation (May) months from 2018–2019 in Southwest Bangladesh and analyzed for complete water chemistry. Selenium (Se) and arsenic (As) were elevated above WHO guidelines in 50% and ~ 87% of samples, respectively, but do not show any recognizable spatial patterns. Shrimp pond and tidal channel water compositions in the dry season (May) are similar, illustrating their connectivity and minimal endogenous effects within shrimp ponds. Tidal channels are less saline in July than shrimp ponds still irrigated by tidal channels, suggesting that either farmers limit irrigation to continue farming saltwater shrimp, or the irrigation flux is low and leads to a lag in aquaculture-tidal channel compositional homogenization. δ^18^O and δ^2^H isotopic compositions from samples in May of 2019 reveal tidal channel samples are closer to the local meteoric water line (LMWL) than shrimp pond samples, because of less evaporation. However, evaporation in May shrimp ponds has a minimal effect on water composition, likely because of regular drainage/exchange of pond waters. Dissolved organic carbon (DOC) is positively correlated with both δ^18^O and δ^2^H in shrimp ponds, suggesting that as evaporation increases, DOC becomes enriched. Multiple linear regression reveals that As and Se can be moderately predicted (adjusted R^2^ values between 0.4 and 0.7, p < 0.01) in surface waters of our study with only 3–4 independent predictor variables (e.g., Ni, V and DOC for Se prediction; Cu, V, Ni and P for As prediction). Thus, this general approach should be followed in other regions throughout the world when measurements for certain hazardous trace elements such as Se and As may be lacking in several samples from a dataset.

**Supplementary Information:**

The online version contains supplementary material available at 10.1186/s12932-021-00074-2.

## Introduction

Although there has been much research in Bangladesh on groundwater and contaminants such as arsenic (As) (e.g., [4, 7, 17, 38, 47, 48]), less emphasis has been placed on surface water chemistry, especially in Southwest Bangladesh. Multiple studies in Bangladesh have geochemically examined tidally dominated rivers (hereafter called tidal channels) and adjacent waterways in both temporal and spatial ways (e.g., [2, 3, 24, 33, 59, 63]). Studies have also looked at trace element concentrations in aquaculture ponds near the coastal region of Bangladesh [23, 32, 62] or in coastal Bangladesh rivers [36, 52] and nonspecified lakes/ponds [1]. However, these studies have focused more on risk assessment, general reporting of trace element or major element concentrations, and overall water quality. While reporting concentrations and changes in water chemistry is useful for preliminary work, more detailed understanding of surface water geochemical relationships is imperative for predicting where waters may have elevated concentrations of certain hazardous elements such as As or selenium (Se), and what ultimately controls the element concentrations.

**Fig. 1:**
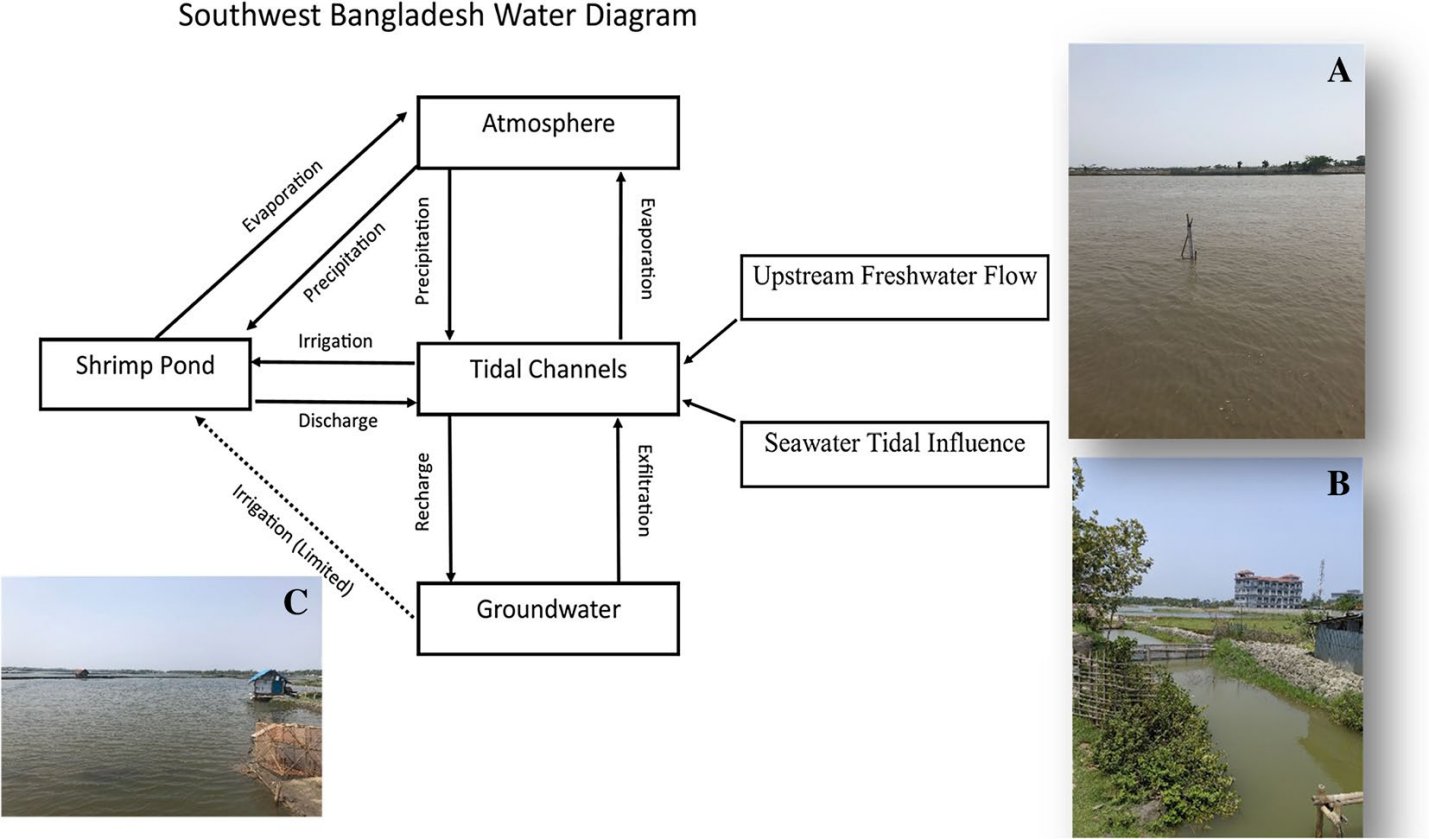
Conceptual diagram of the water cycle within Southwest Bangladesh and example images of tidal channels that irrigate shrimp ponds (both a typical main channel like that sampled in this study (**A**) and an inlet from a smaller, connective channel (**B**). A typical shrimp pond (**C**). A video of tidal channel irrigation to a shrimp pond is provided as Additional file 2

Studies thus far also have not thoroughly researched the relationship of tidal channel waters with aquaculture shrimp ponds, which are often irrigated with tidal channel water during the dry season in Southwest Bangladesh instead of groundwater [6] (Fig. 1). Recent studies indicate that tidal channel waters often have elevated arsenic, especially in the dry season [6] [25], so there is concern that shrimp ponds and the shrimp grown in them may also have high arsenic.

Particularly reliant upon surface water in Southwest Bangladesh (e.g., Satkhira and Khulna districts) are aquaculture and agriculture, where rice farming, fishing, and shrimp farming are the primary sources of income for people in the area (e.g. [11]) and shrimp farming in particular has increased dramatically since the 1980s [9]. Shrimp ponds (bottom left of Fig. 1) are typically 1 m or less in depth on average and vary largely in area, although averaging around 3 ha, while the tidal channels irrigating them have variable depths ranging up to several meters.

In general, critically understanding relationships between trace elements in surface waters in Bangladesh is particularly needed because trace element chemistry in Asian rivers is poorly understood [28], and the aquaculture shrimp ponds and tidal channels in Southwest Bangladesh are an innate offshoot of the large Ganges River system. Additionally, better understanding of water chemistry in Bangladesh aquaculture environments has worldwide applicability to coastal aquaculture systems in other countries such as India and Taiwan. Thus, this study aims to holistically examine possible influences (i.e., precipitation, evaporation, irrigation) on the composition of tidal channel and shrimp pond water in Southwest Bangladesh, and to use multivariate techniques to assess whether As and Se concentrations can be predicted in surface waters. The main research questions are: (1) Do early monsoonal rains affect water compositions of shrimp ponds and tidal channels differently? (2) Are any elements or other geochemical parameters sensitive to endogenous changes within shrimp ponds following tidal channel irrigation? and (3) Can Se and As surface water concentrations be reasonably explained and modeled by a small subset of other variables through multiple linear regression modeling?

## Methods and study area

### Study area

**Fig. 2:**
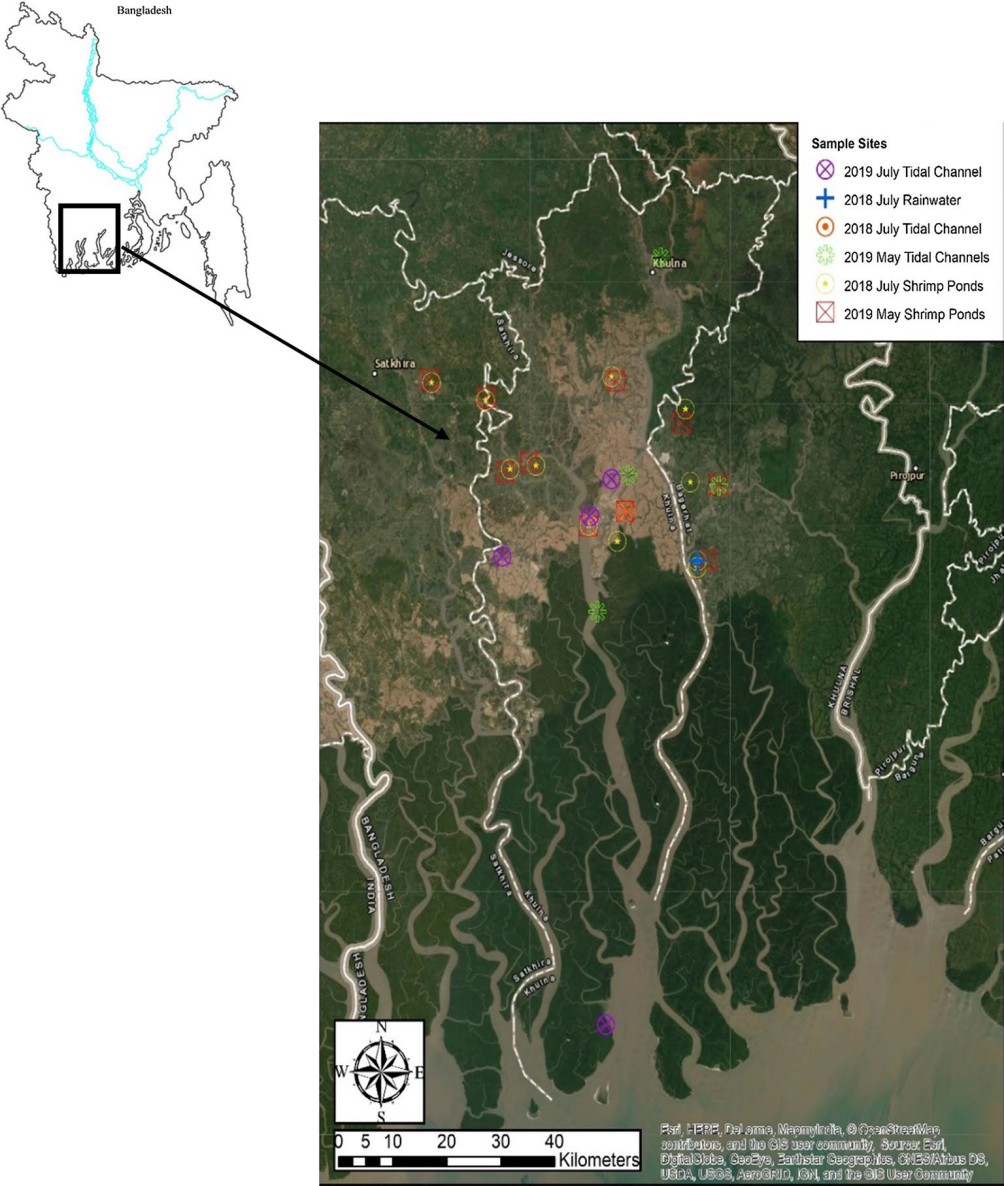
Map of the study area and the sample sites in Southwest Bangladesh, with the dark green area representing the Sundarbans natural mangrove forest

Southwest Bangladesh is a tidally influenced area near the coast of the Bay of Bengal (Fig. 2), with the tidal influence extending just north of Khulna City in the dry season (e.g., [16]). Studies in the area have shown that tidal influence on tidal channel water composition varies by season and the amount of freshwater discharge from upstream [53, 54]. Specifically, salinity ranges between 0 and 2 parts per thousand (ppt) between the tides, and pH ranges 0–0.4 units between tides throughout the year depending on location within the Sundarbans [53, 54]. This tidal area has not been as extensively studied as the northern floodplains in the G-B-M delta or the Himalayan foothills, where river water and sediment compositions were previously researched (e.g., [14, 43, 61, 64]). The tidal region includes part of the Sundarbans mangrove forest, although much of this forest was converted to agricultural islands back in the 1960s–1970s (e.g., [51] and the references cited therein). These agricultural islands are also known as “polders,” and are surrounded by embankments, shielding these islands from storm surges or tidal inundation [5, 51].

Bangladesh experiences a strong monsoonal climate, where biseasonal precipitation causes approximately 80% of yearly rainfall to occur between the months of June to September (e.g., [11, 18, 51]). This large seasonal difference in precipitation can lead to vast changes in surface water chemistry during different seasons, particularly in coastal regions (e.g., [2, 59, 63]).

### Sample collection

Surface water samples were collected in both the early monsoon season in July of 2018 and 2019, and during the end of the dry season in May of 2019. July average precipitation in Khulna is ~ 363 mm, while May average precipitation is ~ 180 mm [10]. All samples were taken within the general vicinity of Southwest Bangladesh, and predominantly within the Khulna District (Fig. 2). All but one shrimp pond aquaculture sample (KA-10) were irrigated with tidal channel water.

Samples were collected as follows:

- **July**: 11 shrimp ponds + 5 tidal channel samples + 1 rainwater sample (n = 17)
- **May**: 10 shrimp ponds + 4 tidal channel samples (n = 14)

May and July shrimp pond samples were taken in the same relative locations to avoid differences in tidal influence and thus allow more direct comparison. Tidal channel samples were taken at different locations between seasons, but those used for dissolved ion concentration comparison in spider diagrams (P32-TC, P32-1 and P32-2 in July; MD-TC-16, MD-TC-18, MD-TC-19, and MD-TC-22 in May) were collected in channels that irrigated the studied shrimp ponds in both seasons. One rainwater sample was collected in July of 2018 from a tin roof collection device.

Surface water samples were collected with plastic syringes and buckets from the upper 1 m of the water column and were rinsed at least once between each sample. Samples were filtered in the field with a syringe and 0.45 µm polypropylene filter into 60- and 125-mL bottles. May 2019 and July 2019 samples were all filtered into 60 mL bottles until the bottles were completely filled to prevent oxygen and hydrogen isotope exchange with headspace before analysis. Only 2019 samples were collected for isotopic analysis because in our lab, analytical capabilities for oxygen and hydrogen isotopes were not yet available in 2018. The July 2018 tidal channel and rainwater samples were placed in 500 mL bottles, with the tidal channel sample filtered in the laboratory with a 0.45 μm polypropylene filter (not filtered in the field).

### Field measurements

A Hach Hydrolab DS5 was used to gather in situ surface water measurements of pH, oxidation–reduction potential (ORP) in millivolts (mV), specific conductivity (SpC) in microsiemens per centimeter (µS/cm), and temperature in degrees Celsius (˚C) for July 2018 shrimp pond, May 2019 shrimp pond, and May 2019 tidal channel samples following the methods of Ayers et al. [6]. July 2018 tidal channel SpC was measured with a hand-held SpC Hanna probe while one July 2018 shrimp pond sample (KA-1) SpC value was estimated through total ion summation.

ORP measurements from the Hach Hydrolab DS5 were used for distinguishing oxic and anoxic conditions for surface water samples, as described in [6, 7]. ORP (relative to the Ag/AgCl redox couple) was converted to Eh (relative to the standard hydrogen electrode (SHE)) in Table 1 by adding 187 mV to each field measurement.

**Table 1:** Sample location information, in-situ measurements, and saturated minerals in solution

Sample	Type	Latitude	Longitude	Date-Time^a^	CIB (%)^b^	Saturated minerals^d^	pH	SpC (µS/cm)^e^	Eh (mV)^g^
KA-5-Blank	Blank								
KA-7-Blank	Blank								
KA-17-Blank	Blank								
KA-20-Blank	Blank								
KA-1	Shrimp Pond July	22.47861853	89.47911456	7/6/2018	0.65%	Kfs, kln, Ms, Dol, Cal, Ilt	8.09	28403.0	399.0
KA-2	Shrimp Pond July	22.50075958	89.43157628	7/7/2018	− 3.61%	Kfs, Ms, Dol, Cal, Ilt	8.50	20200.0	357.0
KA-3	Shrimp Pond July	22.69571769	89.16967095	7/8/2018	− 3.41%	Ms, Dol, Cal	9.00	6541.0^f^	377.0
KA-4	Shrimp Pond July	22.67178417	89.25978431	7/8/2018	− 2.54%	Kfs, kln, Ms, Dol, Cal, Ilt	8.26	20398.0	407.0
KA-5	Shrimp Pond July	22.57789021	89.30023656	7/9/2018	1.63%	Ms, Dol, Cal	8.61	29595.0	390.0
KA-6	Shrimp Pond July	22.58252190	89.34350180	7/9/2018	2.03%	Kfs, kln, Ms, Dol, Cal, Ilt	8.44	28264.0	391.0
KA-7	Shrimp Pond July	22.44306661	89.61203278	7/10/2018	− 3.90%	Dol	8.08	12203.0	600.0
KA-8	Shrimp Pond July	22.45236994	89.61284144	7/10/2018	− 3.87%	Kfs, kln, Ms, Dol, Cal, Ilt	8.38	9053.0	445.0
KA-9	Shrimp Pond July	22.55983540	89.60069553	7/10/2018	− 1.71%	Ms, Dol, Cal	8.19	16466.0	437.0
KA-10	Shrimp Pond July	22.65947095	89.59243177	7/11/2018	− 1.44%	Dol, Cal	9.28	1505.0	389.0
KA-11	Shrimp Pond July	22.70397053	89.46984816	7/11/2018	− 0.83%	Dol, Cal	8.41	22440.0	436.0
P32-TC	Tidal Channel July	22.51957700	89.49199400	7/6/2018	8.00%			6990.0	
KA-8RW	Rainwater	22.45236994	89.61284144	7/10/2018	− 1.16%				
KA- 12	Shrimp Pond May	22.49975329	89.43115749	5/6/19 15:42	7.48%	Kfs, Ms, Dol, Cal, Ilt	8.65	27731.0	390.0
KA- 13	Shrimp Pond May	22.51984753	89.49248609	5/7/19 9:37	8.46%	Kfs, kln, Ms, Dol, Cal, Ilt	8.05	31367.0	431.0
KA- 14	Shrimp Pond May	22.45304998	89.62798504	5/8/19 10:00	4.34%	Kfs, kln, Ms, Dol, Cal, Ilt	7.97	26077.0	414.0
KA- 15	Shrimp Pond May	22.55608476	89.64831928	5/8/19 12:30	4.33%	Kfs, kln, Ms, Dol, Cal, Ilt	8.15	25749.0	418.0
MD-TC-22	Tidal Channel May	22.55505854	89.64806772	5/8/19 12:50	3.33%	Kfs, kln, Ms, Dol, Ilt	7.67	25339.0	454.0
KA- 16	Shrimp Pond May	22.57374567	89.29286777	5/9/19 10:10	4.66%	Ms, Dol, Cal	8.77	28851.0	404.0
KA- 17	Shrimp Pond May	22.58665928	89.33286479	5/9/19 11:25	6.70%	Kfs, kln, Ms, Dol, Cal, Ilt	7.96	33034.0	443.0
KA- 18	Shrimp Pond May	22.67498980	89.26152243	5/9/19 14:00	4.79%	Kfs, kln, Ms, Dol, Cal, Ilt	8.55	11285.0	403.0
KA- 19	Shrimp Pond May	22.69974406	89.16632039	5/10/19 10:10	7.03%	Kfs, Ms, Dol, Cal	8.86	17557.0	359.0
KA- 20	Shrimp Pond May	22.69793983	89.47640710	5/11/19 9:00	3.90%	Kfs, kln, Ms, Dol, Cal, Qz, Ilt	7.77	32046.0	474.0
KA- 21	Shrimp Pond May	22.64063033	89.58598250	5/11/19 11:00	6.41%	Kfs, Ms, Dol, Cal, Ilt	8.61	26270.0	430.0
MD-TC-16	Tidal Channel May	22.38292227	89.44586719	5/6/2019 10:55	4.18%	Kfs, kln, Ms, Dol, Ilt	7.56	36334.0	450.0
MD-TC-18	Tidal Channel May	22.56935935	89.49654172	5/7/2019 10:55	4.07%	Kfs, kln, Ms, Dol, Ilt	7.64	29662.0	458.0
MD-TC-19	Tidal Channel May	22.86607737	89.55133013	5/7/2019 15:27	4.13%	Kfs, kln, Ms, Dol, Ilt	7.63	25837.0	445.0
Hiron Point	Tidal Channel July	21.81744000	89.46000000	7/22/2019 10:05	10.53%				
Site-1	Tidal Channel July	22.45839000	89.28765000	7/25/2019 19:55	9.34%				
P32-1	Tidal Channel July	22.51326000	89.43369000	7/26/2019 8:10	10.47%				
P32-2	Tidal Channel July	22.56404000	89.46957000	7/26/2019 14:50	3.74%				
*Pond and Tidal Channel Geometric Mean*					4.60%^c^		8.27	19842.57	421.69
*Pond and Tidal Channel Std Dev*							0.45	8959.77	48.00

^a^Bangladesh Standard Time (BST or GMT + 6)

^b^Charge imbalance error (%) between major ionic species in solution

^c^Arithmetic mean of absolute value (%)

^d^Saturated minerals have saturation index > 0 (log(Q/K))—Kfs K-feldspar; Kln kaolinite; Ms muscovite; Dol dolomite; Cal calcite; Qz quartz; Ilt illite (Note: No Fe-minerals because of no reported Fe)

^e^Acronym represents specific conductivity (SpC)

^f^Field measurement likely inaccurate based on major ion concentrations

^g^Eh is relative to the standard hydrogen electrode (SHE)

### Sample preparation and analyses

2–10 mL of each filtered sample was acidified with a drop of concentrated nitric acid (HNO_3_) in the laboratory for inductively coupled plasma (ICP) analysis, while approximately 20–30 mL of sample was left unacidified and used for ion chromatography (IC) and total organic carbon analysis (TOC). Approximately 2 mL of filtered, unacidified May and July 2019 samples were transferred to glass vials via plastic pipette immediately upon opening the 60 mL bottles for δ^18^O and δ^2^H isotope analysis.

All acidified water samples were analyzed on a Perkin Elmer NexION 2000B ICP-MS in both standard and kinetic energy discrimination (KED) modes using EPA Method 6020B at Vanderbilt University for the elements As, Be, Cd, Co, Cr, Cu, Fe, Mn, Mo, Ni, Pb, Sb, Se, Ti, Tl, V, and Zn. All acidified surface water samples were also analyzed on an Agilent 5110 VDV ICP-OES using EPA Method 6010D at Vanderbilt University to report the ions: Al, As, B, Ba, Ca, Fe, K, Mg, Na, P, S, Si, and Sr. All 2018 filtered, unacidified surface water samples were analyzed for inorganic and organic carbon content with a Shimadzu model TOC-V CPH/CPN using ASTM Method D-7573-09 at Vanderbilt University, as described in [6, 7], while samples from 2019 were all analyzed via a Shimadzu model TOC-LCPH using EPA Method 9060A. Unacidified water samples were also analyzed for Cl, F, Br, NO_3_, PO_4_, and SO_4_ with a Metrohm 881 Compact IC Pro using SW-846 EPA Method 9056. NO_3_, PO_4_ and F were omitted from the reported results because nearly all values were < MDL.

May and July 2019 water samples were run on a Picarro L2130-i isotope and gas concentration analyzer at Vanderbilt University to provide δ^18^O and δ^2^H isotope values relative to Vienna Standard Mean Ocean Water (VSMOW). Each sample was run twice, with the mean of four measured injections (following four preparatory injections) used to generate δ^18^O and δ^2^H isotope values for each sample run. Average values of the two sample runs are reported. Average precision (1σ) between replicate sample runs was ~ 0.1‰ for δ^2^H and ~ 0.02‰ for δ^18^O. Salt liners were used to prevent damage from salt precipitates. USGS standards USGS45, USGS49 and USGS50 were used to correct measured sample values through the USGS LIMS for Lasers data reduction scheme.

More details on sample preparation are included in the Additional file 1.

### Quality control

Method blanks of samples were taken with deionized water in the field in July 2018 and May 2019 and analyzed for complete chemical analysis (IC, TOC, ICP-MS, ICP-OES), and routinely show concentrations at or below method detection limits (Table 2). For samples run on the ICP-MS, ICP-OES, IC and TOC analyzers, periodically measured concentrations in standards were required to be within 15% of the known value and blanks were required to be below the method detection limit (MDL). A duplicate shrimp pond sample from May 2019 showed all concentrations except Mn in very close alignment with one another (3.0% average difference in concentrations for all reported elements (including DIC and DOC) that had concentrations > 10 ppb), illustrating the representative nature of each sample. Method detection limits are listed in Table 2. Average charge-balance error was 4.6%, similar to the average charge-balance error in [6] of 3.9%.

**Table 2:** Measured geochemical parameters

MDL (mg/L)		0.0034	0.00013	0.00026	0.00016	0.00011	0.00026	0.00020	0.00045	0.00018	0.00031	0.0023	0.0016	0.0065
Sample	Type	Al^1^	V	Cr	Mn	Co	Ni	Cu	Zn	As	Se	B	Ba	Ca
KA-5-Blank	Blank	0.004	0.000	0.001	0.000				0.003	0.000	0.005	0.171		
KA-7-Blank	Blank	0.001	0.000	0.000	0.000				0.003			0.085	0.002	0.16
KA-17-Blank	Blank		0.000	0.004	0.003	0.0003	0.005		0.002	0.004		0.503	0.002	
KA-20-Blank	Blank		0.000	0.003	0.000		0.000		0.003	0.006		0.500	0.000	
KA-1	Shrimp Pond July	0.160	0.008	0.010	0.006	0.0013	0.011	0.018	0.007	0.034	0.076	2.43	0.16	174.62
KA-2	Shrimp Pond July	0.152	0.008	0.009	0.001	0.0009	0.009	0.016	0.006	0.043	0.076	1.83	0.22	168.61
KA-3	Shrimp Pond July	0.250	0.013	0.008	0.007	0.0009	0.007	0.016	0.006	0.036	0.073	1.56	0.27	143.92
KA-4	Shrimp Pond July	0.145	0.016	0.010	0.005	0.0011	0.011	0.021	0.005	0.095	0.095	1.68	0.28	178.91
KA-5	Shrimp Pond July	0.242	0.021	0.013	0.002	0.0016	0.024	0.019	0.006	0.015	0.091	2.28	0.25	212.77
KA-6	Shrimp Pond July	0.228	0.020	0.013	0.013	0.0018	0.024	0.013	0.005	0.018	0.198	1.57	0.19	227.05
KA-7	Shrimp Pond July	0.134	0.004	0.008	0.003	0.0008	0.006	0.012	0.005	0.017	0.074	0.89	0.16	119.44
KA-8	Shrimp Pond July	0.073	0.007	0.006	0.049	0.0005	0.000	0.019	0.006	0.059	0.069	0.56	0.11	93.10
KA-9	Shrimp Pond July	0.121	0.009	0.008	0.000	0.0008	0.009	0.017	0.004	0.007	0.119	1.16	0.25	122.30
KA-10	Shrimp Pond July	0.066	0.005	0.004	0.001	0.0002	0.007	0.009	0.004	0.001	0.060	0.12	0.10	40.87
KA-11	Shrimp Pond July	0.155	0.007	0.010	0.001	0.0011	0.014	0.018	0.006	0.003	0.168	1.58	0.14	176.36
P32-TC	Tidal Channel July	0.016			0.000	0.0001	0.002	0.010	0.000	0.019	0.014	0.47	0.07	70.25
KA-8RW	Rainwater	0.003	0.000		0.004				0.760	0.000	0.004		0.00	1.54
KA- 12	Shrimp Pond May	0.093	0.022	0.004	0.005	0.0004	0.006	0.008	0.007	0.039	0.035	3.40	0.24	237.80
KA- 13	Shrimp Pond May	0.092	0.021	0.003	0.004	0.0003	0.004	0.007	0.003	0.033	0.034	3.56	0.19	247.80
KA- 14	Shrimp Pond May	0.093	0.017	0.006	0.004	0.0004	0.006	0.005	0.003	0.033	0.031	2.90	0.26	232.70
KA- 15	Shrimp Pond May	0.093	0.017	0.005	0.002	0.0003	0.006	0.007	0.004	0.030	0.015	2.94	0.30	214.40
MD-TC-22	Tidal Channel May	0.094	0.018	0.004	0.004	0.0004	0.006	0.006	0.004	0.043	0.012	2.50	0.25	198.60
KA- 16	Shrimp Pond May	0.093	0.019	0.005	0.004	0.0003	0.006	0.004	0.004	0.032	0.029	3.10	0.26	221.90
KA- 17	Shrimp Pond May	0.094	0.019	0.004	0.004	0.0004	0.006	0.007	0.007	0.025	0.034	3.54	0.22	249.60
KA- 18	Shrimp Pond May	0.085	0.011	0.005	0.004	0.0004	0.007	0.004	0.003	0.027	0.035	1.47	0.24	122.30
KA- 19	Shrimp Pond May	0.086	0.018	0.004	0.004	0.0002	0.003	0.005	0.003	0.049	0.037	2.15	0.19	141.80
KA- 20	Shrimp Pond May	0.100	0.020	0.004	0.003	0.0003	0.004	0.005	0.004	0.041	0.054	3.29	0.22	266.80
KA- 21	Shrimp Pond May	0.092	0.019	0.005	0.084	0.0004	0.000	0.003	0.004	0.026	0.029	2.52	0.25	219.50
MD-TC-16	Tidal Channel May	0.104	0.021	0.004	0.003	0.0003	0.006	0.005	0.018	0.038	0.056	3.69	0.11	264.70
MD-TC-18	Tidal Channel May	0.114	0.019	0.005	0.002	0.0004	0.007	0.005	0.011	0.042	0.015	2.85	0.25	223.00
MD-TC-19	Tidal Channel May	0.090	0.018	0.005	0.004	0.0004	0.007	0.006	0.010	0.039	0.030	2.57	0.30	204.60
Hiron Point	Tidal Channel July	0.140	0.017	0.013	0.005	0.0009	0.013	0.017	0.014	0.020	0.101	3.48	0.07	184.21
Site-1	Tidal Channel July	0.182	0.023	0.018	0.003	0.0016	0.019	0.026	0.029	0.041	0.055	3.56	0.09	267.85
P32-1	Tidal Channel July	0.039	0.004	0.009	0.001	0.0002	0.000	0.016	0.008	0.054	0.039	0.82	0.06	44.55
P32-2	Tidal Channel July	0.039	0.001	0.009	0.000	0.0001	0.002	0.021	0.006	0.006	0.008	0.41	0.03	25.72
*Pond and Tidal Channel Geometric Mean*		0.101	0.012	0.0066	0.003	0.0005	0.005	0.010	0.005	0.025	0.045	1.75	0.17	155.51
*Pond and Tidal Channel Std Dev*		0.056	0.007	0.004	0.017	0.0005	0.006	0.006	0.006	0.019	0.045	1.09	0.08	70.12

MDL (mg/L)		0.0032	0.0035	0.0056	0.0068	0.0058	0.0038	0.0037	0.0101	0.0064	0.0254		0.15	0.18
Sample	Type	K	Mg	Na	P	S	Si	Sr	Cl	Br	SO_4_	HCO_3_	DIC*	DOC*
KA-5-Blank	Blank	0.096	0.389	2.27	0.075	0.539		0.002	0.768		0.93		0.450	2.85
KA-7-Blank	Blank	0.093	0.189	2.65	0.032	0.252			0.915		1.23		0.470	2.09
KA-17-Blank	Blank	0.032	0.037	1.93	0.012			0.019					0.272	0.47
KA-20-Blank	Blank	0.024	0.029	0.60									0.225	0.58
KA-1	Shrimp Pond July	279.68	541.62	5279.52	0.089	450.94	0.95	3.66	9250.22	35.42	1108.30	151.60	29.84	5.71
KA-2	Shrimp Pond July	186.15	407.08	3798.48	0.100	352.72	0.70	2.84	7321.86	29.82	860.10	152.90	30.10	8.42
KA-3	Shrimp Pond July	149.33	366.38	3388.44	0.112	284.38	0.57	2.55	6516.78	28.48	707.86	137.60	27.09	10.30
KA-4	Shrimp Pond July	179.83	415.85	3812.76	0.070	335.78	2.01	2.91	7224.22	30.46	830.56	193.00	38.01	8.38
KA-5	Shrimp Pond July	289.88	622.51	6033.30	0.021	525.91	0.28	4.32	10,412.60	38.84	1251.36	93.76	18.46	9.10
KA-6	Shrimp Pond July	218.08	609.45	5270.34	0.086	461.14	1.38	3.91	9182.44	36.72	1108.48	167.50	32.98	22.0
KA-7	Shrimp Pond July	91.32	228.58	1976.76	0.080	202.47		1.59	3920.46	22.42	515.96	41.29	8.13	6.56
KA-8	Shrimp Pond July	58.21	176.56	1414.74	0.326	122.60	1.40	1.17	2798.86	20.58	306.78	193.00	38.00	11.82
KA-9	Shrimp Pond July	137.19	330.07	2929.44	0.067	252.14	0.22	2.22	5463.42	26.82	614.10	169.20	33.32	8.15
KA-10	Shrimp Pond July	12.46	31.79	146.68	0.032	21.93	1.59	0.29	283.54	14.92	50.80	131.90	25.97	8.58
KA-11	Shrimp Pond July	205.94	468.59	4312.56	0.059	396.27		3.18	7885.82	31.98	925.68	138.30	27.24	7.20
P32-TC	Tidal Channel July	54.13	124.34	1375.98	0.013	94.68	1.95	0.86	1980.40		258.52	176.60	34.78	5.70
KA-8RW	Rainwater	0.42	0.72	6.41	0.012	0.63	0.25		9.34		1.99	7.97	1.57	2.60
KA- 12	Shrimp Pond May	293.90	631.90	5781.00	0.035	498.20	1.43	4.05	8721.00	51.54	1518.00	122.10	24.04	8.02
KA- 13	Shrimp Pond May	342.60	703.80	6546.00	0.052	551.30	0.91	4.52	9562.00	64.54	1793.00	159.90	31.49	3.77
KA- 14	Shrimp Pond May	271.80	594.30	5532.00	0.026	442.10	1.94	3.76	8895.00	48.85	1444.00	162.20	31.93	4.73
KA- 15	Shrimp Pond May	283.00	597.80	5501.00	0.038	448.10	1.21	3.84	8916.00	45.87	1341.00	157.80	31.07	4.11
MD-TC-22	Tidal Channel May	278.20	567.00	5405.00	0.054	436.30	2.12	3.65	8869.00	44.86	1337.00	146.40	28.82	2.86
KA- 16	Shrimp Pond May	311.60	662.30	6091.00	0.050	501.30	0.60	4.22	9755.00	56.42	1544.00	122.30	24.08	5.28
KA- 17	Shrimp Pond May	372.20	778.10	7148.00	0.030	589.20	1.75	4.98	10,920.00	61.37	1872.00	148.70	29.28	3.19
KA- 18	Shrimp Pond May	101.30	244.10	2353.00	0.049	171.70	2.34	1.64	3733.00	11.14	531.30	179.70	35.38	6.91
KA- 19	Shrimp Pond May	175.50	368.80	3741.00	0.238	231.00	1.00	2.38	5674.00	22.88	724.70	181.60	35.75	15.62
KA- 20	Shrimp Pond May	351.30	763.90	7007.00	0.036	560.00	2.89	4.76	11,380.00	70.65	1822.00	196.20	38.64	5.55
KA- 21	Shrimp Pond May	265.10	630.70	5718.00	0.021	445.20	1.55	3.76	8926.00	48.64	1347.00	94.31	18.57	7.60
MD-TC-16	Tidal Channel May	422.80	865.10	7938.00	0.065	671.00	1.48	5.52	12,810.00	88.36	2081.00	143.80	28.31	2.36
MD-TC-18	Tidal Channel May	331.20	668.80	6378.00	0.027	518.40	1.91	4.28	10,250.00	62.98	1649.00	145.60	28.67	3.14
MD-TC-19	Tidal Channel May	291.10	589.90	5492.00	0.048	453.30	2.29	3.76	8837.00	47.60	1457.00	150.80	29.69	3.20
Hiron Point	Tidal Channel July	240.62	571.61	7481.70		440.44	2.24	3.69	10,285.90	30.60	1165.50	114.60	22.57	2.74
Site-1	Tidal Channel July	383.72	877.00	10,726.32	0.014	663.41	1.35	5.52	15,269.20	48.40	1765.20	137.40	27.05	3.05
P32-1	Tidal Channel July	22.37	71.70	817.12		51.26	2.90	0.52	1116.12	3.84	137.68	100.70	19.83	3.12
P32-2	Tidal Channel July	3.01	7.15	23.04		4.98	3.16	0.11	32.09	0.13	13.50	95.03	18.71	2.80
*Pond and Tidal Channel Geometric Mean*		158.16	361.47	3254.04	0.050	275.20	1.34	2.47	5350.28	28.51	766.12	138.10	27.19	5.67
*Pond and Tidal Channel Std Dev*		118.31	243.64	2498.31	0.068	187.98	0.77	1.52	3724.62	20.35	587.11	34.91	6.87	4.25

^1^All measurements in mg.L^−1^, including method detection limits (MDL). Omitted values are negative reported values

^*^Acronyms represent dissolved inorganic carbon (DIC) and dissolved organic carbon (DOC)

### Data reduction

Maps were generated using ArcGIS 10.6.1 and QGIS 3.10, while RStudio and Microsoft Excel were used for figure generation and statistical analyses. Multiple linear regression subset selection used the “leaps” package in R, with 8 variables used as the max number of subsets [42]. The Geochemist’s Workbench 14.0 was used to generate a Piper diagram and an evaporation model in the React program, using sample MD-TC-22 as the input basis. HCO_3_^−^ concentrations (from measured dissolved inorganic carbon (DIC)), charge imbalance, and mineral saturation indices were calculated using SpecE8 software in The Geochemist’s Workbench 14.0 with the default thermo.dat database [13]. Uncertainties are reported as sample standard deviation (1σ).

## Results

### Geochemical concentrations

Dissolved concentrations tend to be lognormally distributed, so they are summarized using the geometric mean. Element concentrations reflect relatively saline waters in shrimp ponds regardless of season, shown by high conservative ion concentrations approaching average seawater concentrations (Table 2). Tidal channel waters show more variability in conservative ion concentrations between sampling months compared to shrimp ponds. Eh values in both sampling months are generally consistent and positive in surface waters, indicative of oxidizing conditions (422 ± 48 mV). Including both sampling months (because of minimal seasonal variability in DOC), the mean shrimp pond DOC concentration is 7.3 ± 4.3 mg.L^−1^, while the mean tidal channel DOC concentration is 3.1 ± 1.0 mg. L^−1^, compared to a river world average of 5.8 mg. L^−1^ and 12 mg. L^−1^ median value in the world’s eutrophic lakes [6] and the references therein). pH values show relatively little variability regardless of sampling month, although shrimp pond waters at 8.4 ± 0.4 are markedly more alkaline than tidal channel waters at 7.6 ± 0.1. Out of elements with known adverse health outcomes in excessive quantities, Cr (6.6 ± 3.6 μg. L^−1^) and Mn (3 ± 17 μg. L^−1^) overall surface water values are all well below levels of health concern [74], while Pb, Be, Cd, and Tl were either close to detection limit or below detection limit and thus not reported. Ti, Sb, Fe and Mo values are also not reported because of either low values (e.g., nearly all May samples had negative reported Fe values) or concerns with interference on the ICP. However, both As (25 ± 19 μg. L^−1^) and Se (45 ± 45 μg. L^−1^) are at levels of potential health concern across both sampling months based on WHO and EPA drinking water guidelines, which are 10 μg. L^−1^ for As for both the EPA and WHO, and 50 μg. L^−1^ and 40 μg. L^−1^, respectively, for Se EPA and WHO guidelines [70, 74].

### Element and geochemical parameter correlations

Concentrations of most conservative elements are strongly correlated, indicative of their similar mobilities in solution (Additional file 1: Fig. A1). Concentrations of many elements that tend to behave nonconservatively such as As and Se do not show strong correlations, indicative of redox conditions and sorption/desorption mechanisms possibly affecting concentrations. However, pH and Eh measured in shrimp ponds do not correlate well with any elements (Additional file 1: Fig. A2). DOC, important in groundwater chemical reactions in Bangladesh, does not show strong correlations with any elements (Additional file 1: Fig. A1).

### Monthly composition differences and element enrichment relative to seawater

**Fig. 3:**
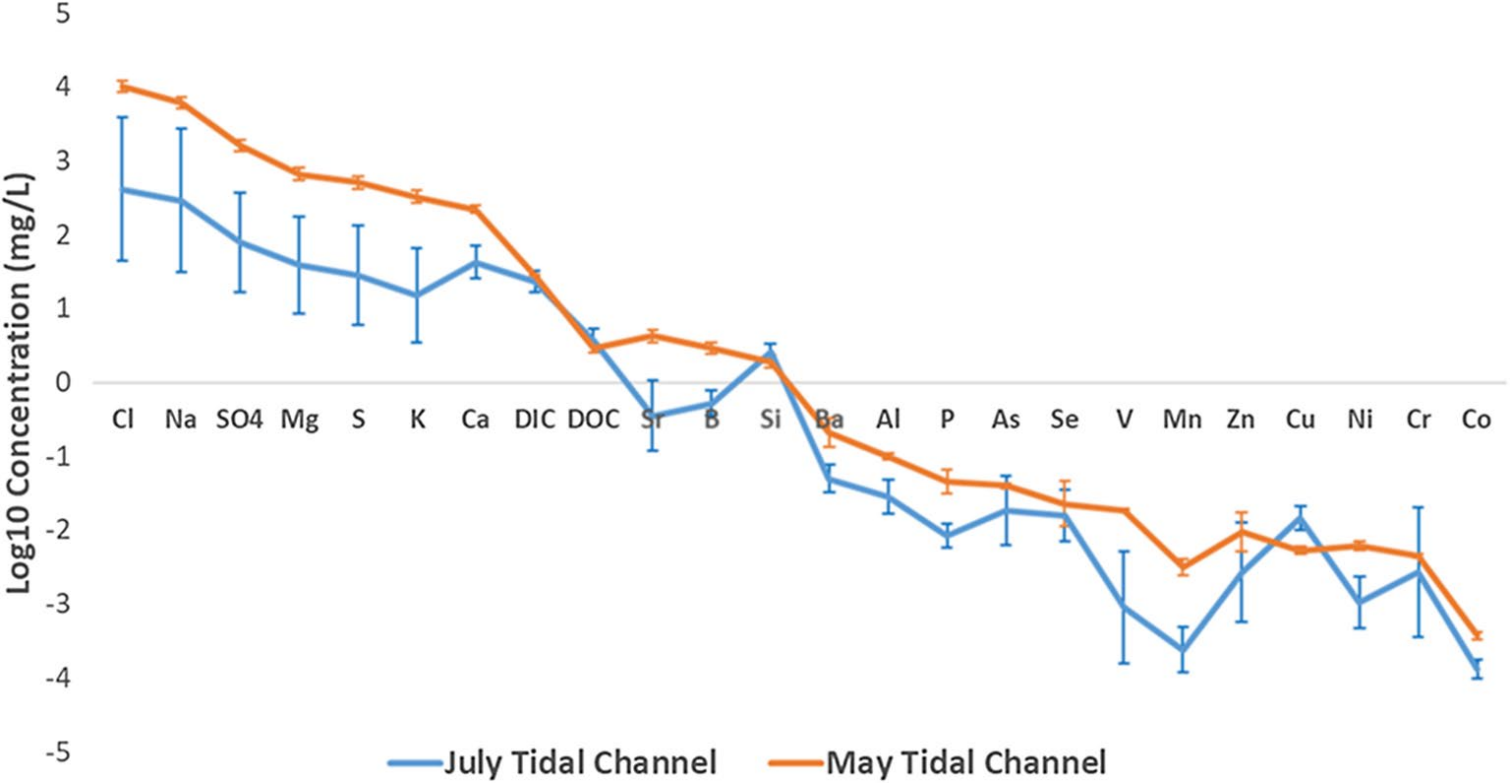
Spider diagram of tidal channel arithmetic mean log10 concentrations, arranged from left to right with decreasing concentrations in seawater. 1σ error bars are given. Illustrates the seasonality between tidal channel samples, with larger variation in July because of early monsoonal rains coupled with tidal influence. July tidal channel samples P32-TC, P32-1 and P32-2 were averaged instead of other July tidal channel samples because of proximity to the shrimp ponds they were irrigating

**Fig. 4:**
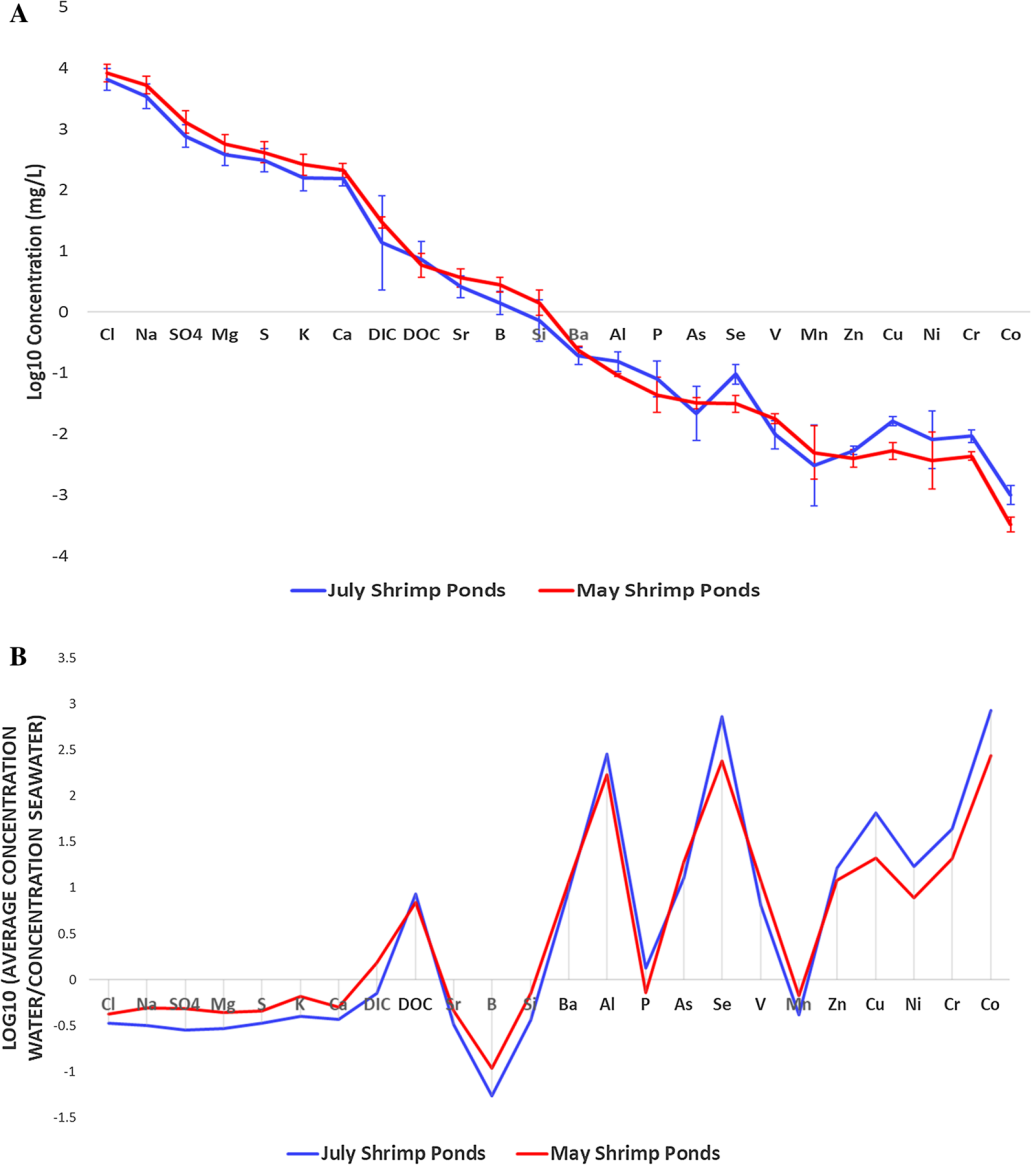
Spider diagrams of arithmetic mean log10 concentrations in July (excluding KA-10) and May shrimp ponds. Slight seasonality is apparent between July and May shrimp ponds irrigated with tidal channels (**A**), albeit with overlap within 1σ error bars. Concentrations in shrimp ponds normalized to seawater (**B**), with log10(geometric mean/seawater) instead of arithmetic mean of log10 concentrations, with points above 0 showing relative enrichment and points below 0 showing relative depletion compared to seawater. Two Si values are omitted from the July shrimp pond data because of anomalous negative values

In general, lower element concentrations are seen in July tidal channel waters relative to May tidal channel waters (Fig. 3). Concentrations in July tidal channel waters, however, show much more variance, although nonconservative elements have less consistency in variation. July and May shrimp ponds show slight differences in average compositions, albeit with most overlap contained within 1σ error bars (Fig. 4a). However, Se, Cu, Cr, and Co are all elevated in July shrimp ponds relative to May shrimp ponds outside of 1σ variation. July shrimp ponds are slightly less saline on average than May shrimp ponds (Additional file 1: Fig. A3). When normalizing shrimp pond (Fig. 4b) and tidal channel (Additional file 1: Fig. A4) concentrations to that of seawater (values from literature; Additional file 1: Table A1), several elements are clearly enriched, such as Ba, As, Se, and Zn. Additionally, DOC is well enriched in shrimp ponds relative to values typically seen in the Indian Ocean [50], shown through DOC plotting well above 0 in Fig. 4a.

### Tidal channel versus shrimp pond composition

**Fig. 5:**
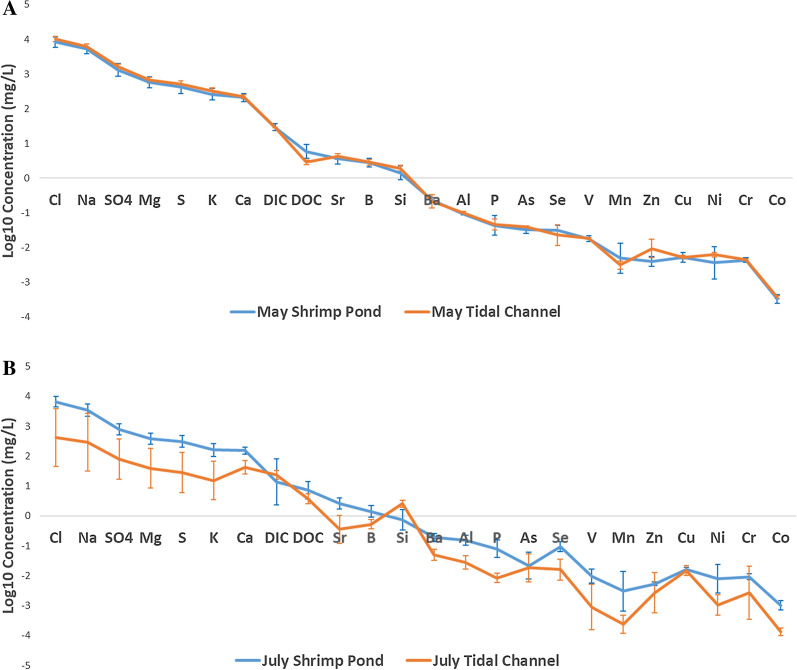
Spider diagrams showing arithmetic mean log10 concentrations in shrimp ponds (excluding KA-10) and tidal channel waters. Strong similarity is seen between May shrimp ponds and the tidal channels irrigating them (**A**). Less similarity is seen in July (**B**), with large variance seen in July tidal channels and lack of overlap between the two water types. Two Si values are omitted from the July shrimp pond data because of anomalous negative values

May tidal channels and shrimp ponds have similar average compositions, although there is more variability in the nonconservative elements such as Zn and Ni (Fig. 5a). In July however, tidal channel and shrimp pond compositions are not as similar, although it is noted that there is high variability within July tidal channel samples (Fig. 5a). In general, July shrimp ponds have higher average concentrations of elements and DOC compared to July tidal channels.

### Oxygen and hydrogen isotopes specific trends

**Fig. 6:**
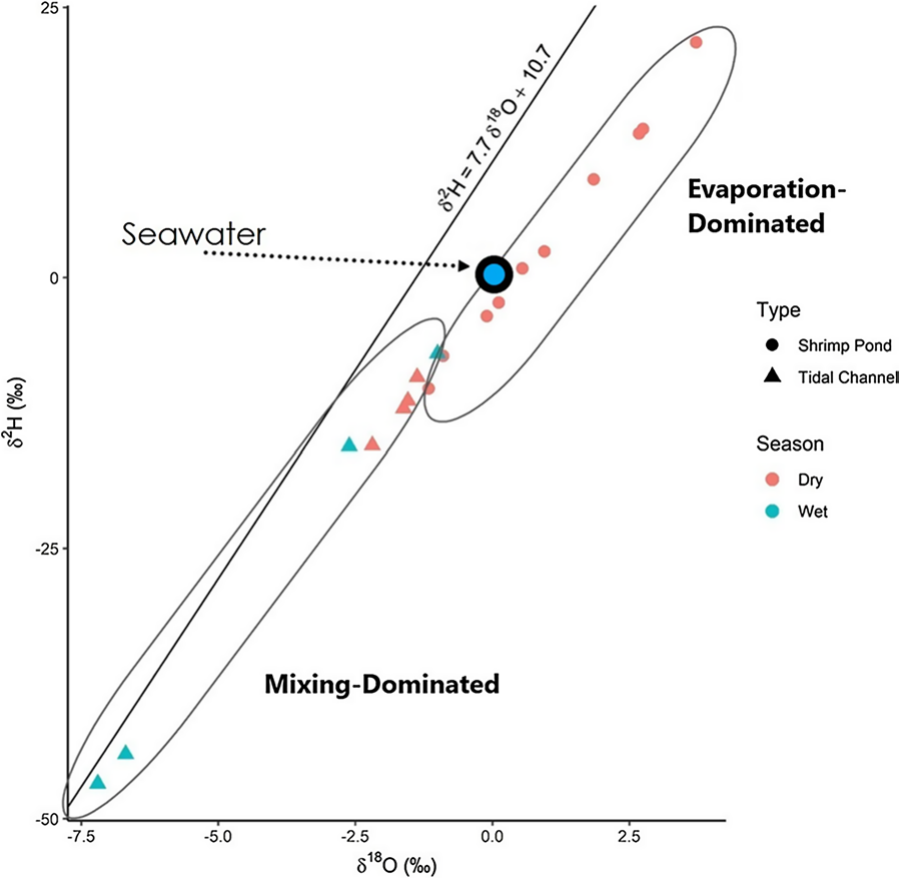
Bivariate plot of δ^18^O and δ^2^H isotopes from May shrimp ponds and May/July tidal channels. Dry season represents samples collected in May, wet season in July. Local meteoric water line (LMWL) taken from [45]. “Seawater” is theoretical seawater at 0‰ δ^18^O and δ^2^H, with a large symbol plotted to represent deviation from exact 0‰ isotopic values in the Bay of Bengal

δ^18^O and δ^2^H from tidal channel samples (Table 3) plot relatively close to the local meteoric water line (LMWL) (Fig. 6). Furthermore, they plot linearly between the LMWL and the expected value for seawater. Shrimp ponds plot farther off the LMWL than tidal channel samples and their stable isotope values are heavier (more positive) than tidal channel isotope values.

**Table 3:** Isotopic measurements relative to VSMOW

Sample	Type	δ^18^O (‰)^VSMOW^	δ^2^H (‰)^VSMOW^
KA- 12	Shrimp Pond May	1.9	9.1
KA- 13	Shrimp Pond May	− 0.1	− 3.6
KA- 14	Shrimp Pond May	0.1	− 2.3
KA- 15	Shrimp Pond May	− 1.2	− 10.3
MD-TC-22	Tidal Channel May	− 2.2	− 15.5
KA- 16	Shrimp Pond May	0.6	0.8
KA- 17	Shrimp Pond May	− 0.9	− 7.3
KA- 18	Shrimp Pond May	2.8	13.8
KA- 19	Shrimp Pond May	3.7	21.8
KA- 20	Shrimp Pond May	1.0	2.4
KA- 21	Shrimp Pond May	2.7	13.3
MD-TC-16	Tidal Channel May	− 1.4	− 9.2
MD-TC-18	Tidal Channel May	− 1.5	− 11.4
MD-TC-19	Tidal Channel May	− 1.6	− 12.1
Hiron Point	Tidal Channel July	− 2.6	− 15.6
Site-1	Tidal Channel July	− 1.0	− 7.0
P32-1	Tidal Channel July	− 6.7	− 44.0
P32-2	Tidal Channel July	− 7.2	− 46.8

^VSMOW^Isotope values are delta values relative to VSMOW (Vienna Standard Mean Ocean Water)

**Fig. 7:**
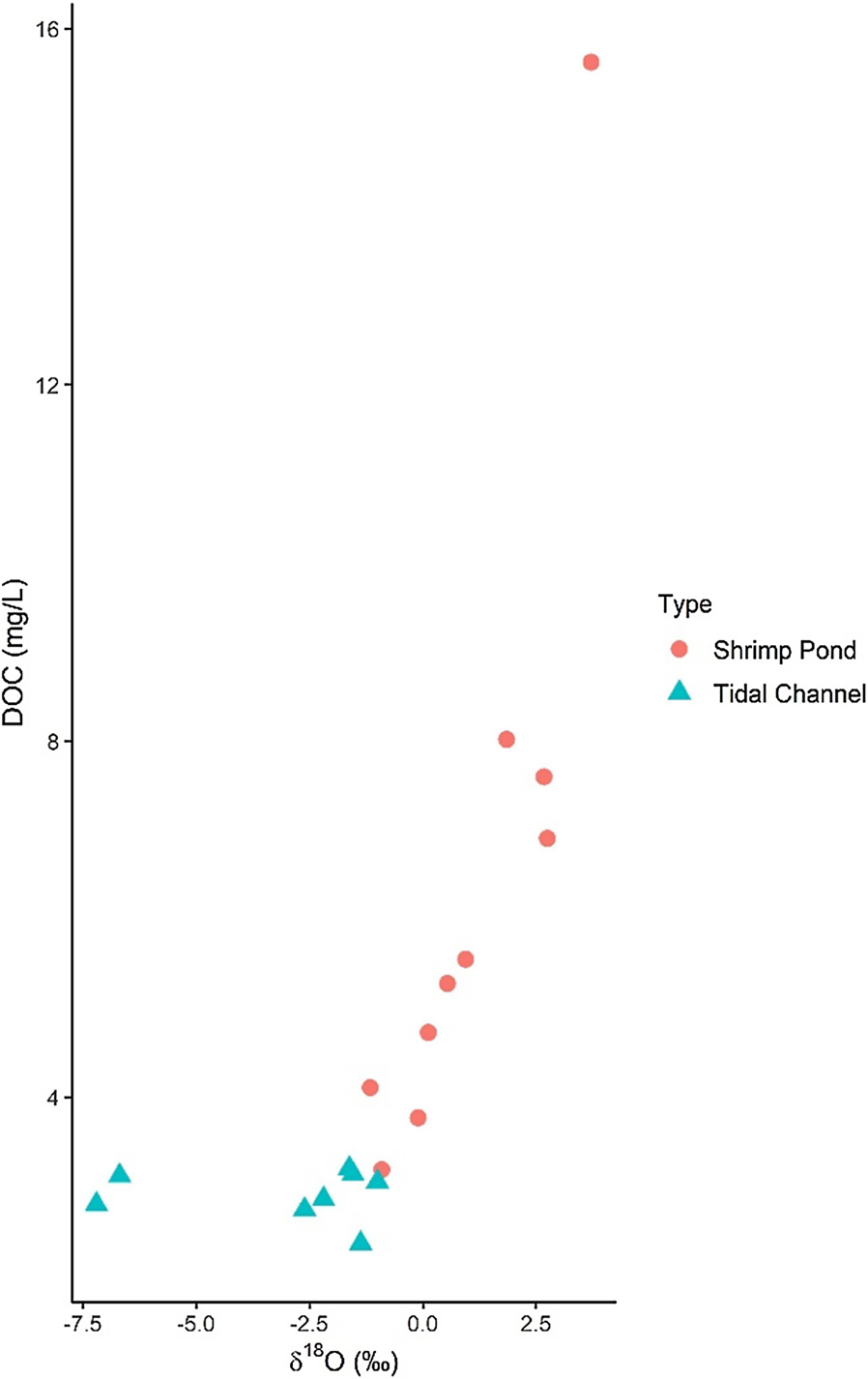
Bivariate plot of δ^18^O from May shrimp ponds and May/July tidal channels showing DOC increasing with enrichment of isotopes in shrimp ponds. Spearman correlation coefficients (DOC is non-normally distributed) of 0.92 between δ^18^O and DOC in shrimp ponds (p = 0.00047) and 0.095 in tidal channels (p = 0.84)

δ^18^O and δ^2^H show a strong positive correlation with DOC in shrimp ponds (δ^18^O and DOC; Spearman ρ = 0.92, p = 0.00047) (Fig. 7). As shrimp ponds become more enriched in both ^18^O and ^2^H isotopes, DOC increases. Tidal channel samples do not show this same relationship (δ^18^O and DOC; Spearman ρ = 0.095, p = 0.84), with DOC values remaining quite stable as δ^18^O and δ^2^H increase.

### Surface water evaporation models

Through the React program in Geochemist’s Workbench (GWB), a simple evaporation model was performed (Additional file 1: Table A2) from the initial 1 kg solution of sample MD-TC-22 to estimate the amount of water evaporation that would yield the concentrations of conservative elements in the irrigated shrimp pond (KA-15) directly adjacent to sample MD-TC-22.

The model estimates that only a relatively small fraction of water evaporation (~ 0.5–10%) is needed to concentrate non-reactive major conservative ions in solution to the concentrations observed in the irrigated shrimp pond (KA-15). When looking at modeled salinity (essentially grouping all major seawater ions together), 1.5–2% evaporation of water can explain the relative increase in salinity observed from tidal channel to shrimp pond water (Additional file 1: Fig. A5), which is close to intersample variability.

In addition to the aforementioned mass-balance focused geochemical model, relatively straightforward isotopic equations can be used to estimate the amount of evaporation in a shrimp pond after tidal channel input. Using several simplifying assumptions such as thermodynamic equilibrium and an equilibrium fractionation factor (α (l/v)) of 1.0098 at 20˚C (even though sampling temperatures were ~ 30–35˚C) for ^18^O/^16^O exchange [44], a Rayleigh distillation equation may be used to estimate the residual fraction of water (f) left in a system (Eq. 1), [29]:

1 $$R = R_{0} \times f^{(1/\alpha - 1)}$$

where R is the isotopic ratio of the residual water and R_0_ is the isotopic ratio of the initial water. In a system where water is evaporating and the liquid phase is known to be more enriched in the heavier stable isotope (i.e., ^18^O), the term for the fractionation factor (α) becomes < 1 (1/α), causing isotopic enrichment in the residual liquid phase as f decreases. After converting the δ^18^O value of the irrigating tidal channel (MD-TC-22) to an ^18^O/^16^O ratio and employing the Raleigh equation, ~ 10% of evaporation of the initial amount of material can explain the more enriched ^18^O/^16^O ratio of the directly irrigated shrimp pond (KA-15). However, this also assumes no other influences on stable isotopes in the shrimp pond, such as precipitation, and assumes that all water was derived from the tidal channel. These are reasonable assumptions, as during the sampling period in May 2019 no measurable precipitation was observed, and the impermeable clays lining most of the ponds prevents groundwater exfiltration or seepage from other water sources [6, 7].

The discrepancy between the estimated 2% evaporation based on salinity and 10% evaporation based on isotopes may be in part due to slight non-conservative processes occurring in ponds such as ion exchange that affect saltwater ions, and our isotopic evaporation model is merely an estimate, as we assume an open system with Rayleigh fractionation. In reality, the system is likely somewhere between an ideal open and ideal closed system. Additionally, fractionation is likely slightly different at the warmer temperatures (> 20˚C) that occurred during our study.

### Selenium and arsenic spatial and seasonal variability

Arsenic concentrations in shrimp ponds and tidal channels are close to or above WHO drinking water guidelines (10 μg. L^−1^) in both May and July samples throughout SW Bangladesh (Additional file 1: Fig. A5). There does not appear to be any spatial correlation between measured arsenic values in both shrimp ponds and tidal channels. Additionally, selenium values are close to or above WHO drinking water guidelines (40 μg. L^−1^) throughout SW Bangladesh in tidal channels and shrimp ponds, with seemingly random spatial concentration trends (Additional file 1: Fig. A7). In general, spatial heterogeneity in surface water trace element compositions appears to be the norm.

While both Se and As are within 1σ variation in May and July tidal channels (Fig. 3), Se is greater in July shrimp ponds compared to May shrimp ponds outside of 1σ variation (Fig. 4a). Arsenic is within 1σ variation when comparing May and July shrimp ponds (Fig. 4a).

### Multiple linear regression

**Fig. 8:**
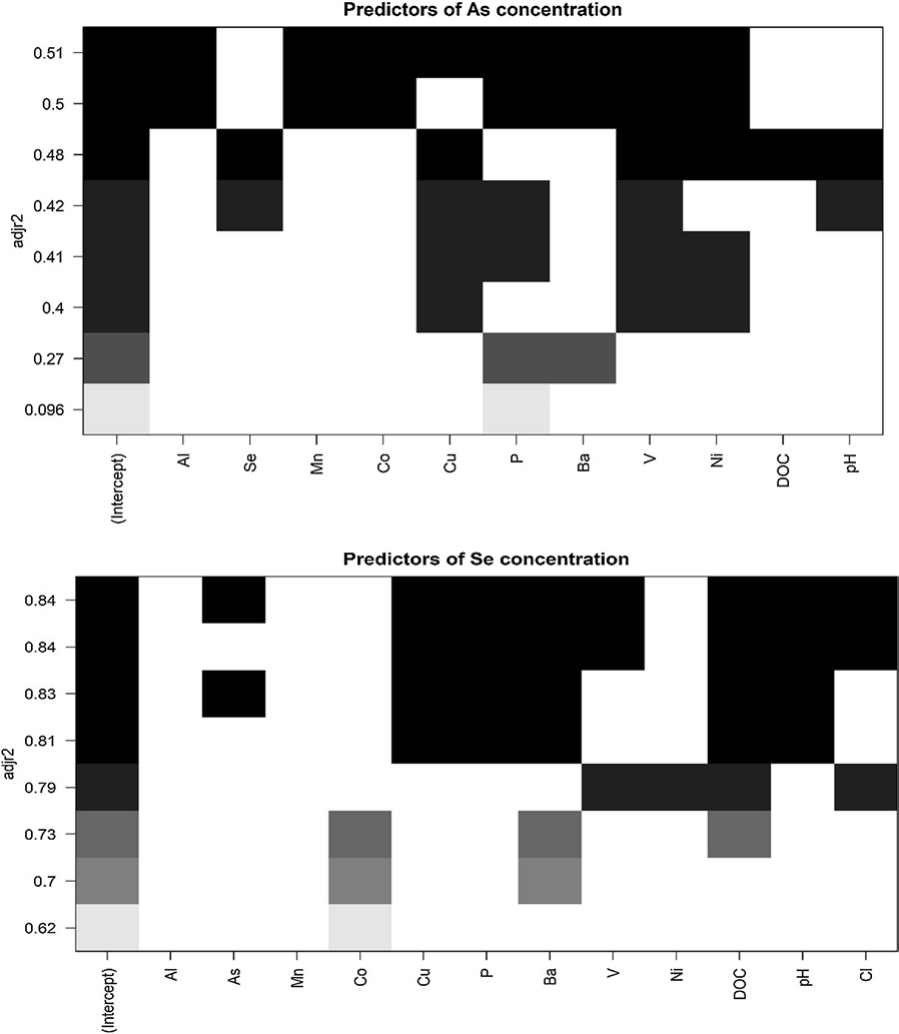
Multiple linear regression models for grouped variables initially selected for Se and As, with adjusted R^2^ values along the y-axis. The best model was computed for each subset size (e.g., each amount of predictor variables utilized in the overall model). n = 25 because of some samples missing predictive variable measurements and thus being omitted, which also explains the slight discrepancy between multiple linear regression analyses completed with more samples (i.e., Additional file 1: Table A4)

Best subsets multiple linear regression (details on variable selection in the Supplementary Document) suggests that the variables Cu, P, V and Ni can explain most As variance (Fig. 8), with an adjusted R^2^ of 0.39 (p = 0.006) (Additional file 1: Table A3). Their significance is supported by V, Ni, and Cu each having p-values < 0.05 when looking at those four variables together as a linear regression fit for As (Additional file 1: Table A3). For Se, linear regression using the concentrations of only V, Ni, DOC, and Cl result in one of the best adjusted R^2^ values (0.74) (Additional file 1: Table A4; Fig. 8), and each variable has a p-value < 0.01 (Additional file 1: Table A4). When removing Cl from the predictive model, the adjusted R^2^ becomes 0.67 (p < 0.0001) with each variable (V, Ni and DOC) having a p-value < 0.01 (Additional file 1: Table A5).

Details of other models and violations of multiple linear regression assumptions are provided in the Additional file 1.

## Discussion

### Trace element enrichment in surface waters

Trace elements of initial focus in this study were Mn and As, because of their known elevated concentrations in groundwater in the region (and potential to enter surface water through groundwater irrigation upstream or exfiltration) and known adverse health effects in excessive quantities (e.g., [27, 34, 56, 58]). Furthermore, [6] measured elevated As > 10 μg. L^−1^ (WHO drinking water guideline, [74]) in 78% of shrimp ponds on Polder 32 in SW Bangladesh (several samples in this present study are from/around the same polder), with 71% of May tidal channels around Polder 32 containing As > 10 μg. L^−1^ as well. [6] also measured Mn > 400 μg. L^−1^ (WHO health-based value, [74]) in 6% of surface water samples.

In this study, however, no dissolved Mn concentrations were above the WHO health-based value of 400 μg. L^−1^ [74] in either shrimp ponds or tidal channels, which is expected based on Mn solubility decreasing when pH is > 6, due to the formation of solid-phase Mn oxides and hydroxides (e.g., [35]). Thus, elevated Mn values were reported in some of [6]’s shrimp pond samples despite similar pH and redox conditions. Arsenic was elevated above the WHO standard in almost all shrimp pond and tidal channel samples regardless of season, with ~ 87% containing As > 10 μg. L^−1^ (Table 2). This supports data from [6] where elevated As in SW Bangladesh surface waters was also reported. However, dissolved As is not expected to be as elevated in near-neutral pH, oxidizing conditions such as those seen in surface waters (e.g., [6, 65]), and thus more research on As cycling at the groundwater-surface water interface in Bangladesh is needed (e.g., [12]).

Arsenic values were > 10 μg. L^−1^ in 75% of July samples (shrimp pond and tidal channels) during the early monsoon in this study, while [6] measured only 11% of tidal channels in October with As > 10 μg. L^−1^. This suggests that the monsoon rainfall has not yet reached a high enough continuous level in July to thoroughly dilute the source of surface water As compared to October. In Khulna the average precipitation in the preceding four months were ~ 1,261 mm in October, only ~ 142 mm in May, and ~ 627 mm in July [10].

Because there were no previous reports of high Se in SW Bangladesh surface waters, it was not an initial element of concern going into this study. Furthermore, Se has been reported as low in Bangladesh groundwater (e.g., [26, 27, 55]), so even if heavy groundwater irrigation or exfiltration could affect surface water concentrations, Se was not expected to be elevated. However, dissolved Se was found to be > 50 μg. L^−1^ (EPA MCL; [70]) in 50% of all samples and > 40 μg. L^−1^ (WHO guideline; [74]) in 50% of all samples (Table 2), with substantially higher average concentrations in the July shrimp ponds compared to May shrimp ponds (Fig. 4a). While low levels of Se intake can lead to nutritional deficiencies in humans such as impaired growth, or thyroid function abnormalities (e.g., [73]), excessive Se intake can be toxic for humans and animals, particularly for inorganic species of Se [68]. However, when shrimp ponds are often converted to rice paddies in the wet season in SW Bangladesh, the chemically reducing paddy sediment likely leads to the formation of insoluble elemental Se [73].

When normalized to average concentrations in seawater, As, Se and Mn are all enriched in shrimp ponds (Fig. 4b), while As and Se are also enriched in tidal channels (Additional file 1: Fig. A4). Thus, the sourcing of these “enriched” elements cannot be explained by seawater from the Bay of Bengal alone, and must have outside sources such as riverine flow, direct anthropogenic input (fertilizers or other chemical supplements), or groundwater flow. The higher Se concentrations in July shrimp ponds versus May shrimp ponds is difficult to explain (Fig. 4a), particularly because the same distinct seasonality is not seen in tidal channels (Fig. 3). A possible explanation is that desorption mechanisms are at work in the July shrimp ponds, although there are no correlations between Se and Eh or pH to suggest this is redox or pH influenced (Additional file 1: Fig. A2). Because colloids can have an important influence on trace elements in solution (e.g., [28]), future spectroscopy and speciation analyses may help identify whether they play an important role in enrichment and seasonality in Bangladesh.

### Tidal channel and shrimp pond connectivity

Strong compositional similarities were seen between May shrimp ponds and the tidal channel sources irrigating them (Fig. 5a). This suggests that there is little compositional change within the shrimp ponds after irrigation, although there is more variance between nonconservative elements, suggesting that sorption/desorption reactions may be occurring after shrimp ponds have been irrigated. This may particularly be true for cation species like Ni and Zn, which may undergo desorption in more saline waters due to competition for sorption sites with salt cations such as Ca and Na (e.g., [60, 66]). Across all dissolved water samples in this current study, moderate positive correlations between metal cations such as Co, Ni, and Cu (Additional file 1: Fig. A1) suggest similar geochemical processes occurring in the natural environment, such as sorption/desorption effects. July shrimp ponds show much less compositional overlap with July tidal channels (Fig. 5b). However, there is high variance of dissolved ion concentrations within July tidal channel samples (Fig. 3), which could be indicative of salinity fluctuation closely tied to rainfall and river discharge. July shrimp ponds and tidal channels may not be as similar compositionally because of shrimp farmers limiting irrigation of less saline waters to prolong harvest of brackish water shrimp varieties, or because of slow mixing rates between the irrigation source (tidal channels) and the shrimp ponds, which may not become complete until late in the monsoon season (i.e., late August or September). Future work analyzing water fluxes in and out of shrimp ponds over time would help better quantify the mixing of pond water and irrigation source.

### Enrichment of As and Se

Arsenic and selenium concentrations vary widely throughout the region. However, they are both ubiquitously elevated > WHO drinking water guidelines in May and July shrimp ponds and tidal channels (Additional file 1: Figs. A6, A7). The widespread occurrence of high As and Se concentrations throughout the region suggests that As and Se are predominantly not locally point-sourced.

Arsenic may be sourced from arsenic-rich groundwater in the region (often > 10 μg. L^−1^) as previously suggested [6], upstream weathering in the Himalayas, or anthropogenic effluent run-off from cities upstream such as Khulna. Because As shows little to no correlation with conservative ions found in seawater and tends to behave nonconservatively (Additional file 1: Fig. A1), it is difficult to deduce whether As increases as more groundwater is present in the system, particularly because there are two possible lower salinity end-members (riverine flow from upstream and groundwater flow) with potentially different As concentrations. It is noted that a January (dry season) freshwater river sample in the nearby Meghna River (where groundwater As concentrations are > 10 μg. L^−1^) had As < 10 μg. L^−1^ [12], although this river has a different drainage basin.

Arsenic concentrations in this study are also much greater than surface waters sampled in 2016 in the same geographic region [8]. The differences in As concentrations between [8] and both this study and [6] may be attributed to small sample size, overall less saline (and thus potentially more diluted) samples in Ayers et al. [8], and sample location heterogeneity, as all three studies used similar methodology. For example, if groundwater exfiltration is a possible source of As to surface waters, sampling locations from [8] may receive different exfiltration rates of groundwater because of widespread geologic heterogeneity in the area (e.g., [7]).

Selenium concentrations higher than average seawater in estuaries can be caused by conservative mixing of polluted/enriched Se river water and relatively Se-depleted seawater, such as in the Solent area in mid-southern England [46] or the Rhone river delta off the Mediterranean Sea [31]. Anthropogenic input of Se was documented in the San Francisco Bay estuary from both sewage treatment plants and oil refineries, albeit the concentrations in the mixed estuary samples were much lower than those seen here [21, 22]. Selenium was also elevated relative to natural background in agricultural irrigation drainage in central California in the mid-1980s [40]. Thus, Se may be sourced from sewage, oil refineries, or agricultural runoff in the area. It is also interesting that Se concentrations are much higher in our samples from 2018 and 2019 (regardless of season) compared to results from surface waters in the same general study area in 2016 analyzed using the same methods [8]. One possible explanation for this is that Se is anthropogenic and the source of its release into the dissolved load began after sampling in 2016, potentially from industrial centers in Khulna [25], while another possible explanation is that the overall less saline samples taken in 2016 were more diluted with rainwater or other sources than ours in 2018 and 2019, thus lowering Se concentrations.

Recently, in the Ganges River system, “hot spots” of elevated trace element concentrations (relative to “background” in the study) were observed near large urban and industrial areas, but these concentrations became diluted by other river tributaries downstream [15]. Thus, a city like Khulna could be a source of Se or other trace elements, and dilution is limited until either the tidal channels empty into the Bay of Bengal or it becomes peak monsoon season. Regardless of the source, a dramatic increase in Se input would be necessary for an increase in surface water concentrations from ~ 0.5–2 μg. L^−1^ in 2016 to ~ 10–200 μg. L^−1^ in 2018–2019.

### Surface water isotopic composition, trends, and evaporative effects

δ^18^O and δ^2^H isotopes in surface waters can be useful proxies for illustrating mixing or evaporative processes. Any samples plotting on the local meteoric water line (LMWL) are theoretically local precipitation, while samples plotting to the right of the LMWL have undergone fractionation, which can be interpreted as evaporation [29], or mixing with isotopically enriched water such as seawater. All tidal channel samples plot close to the LMWL and trend towards the isotopic value of seawater as they become heavier or more enriched, suggesting they have undergone minimal evaporation and represent mixing between rainwater and seawater (Fig. 6). Dry season (May) shrimp pond samples are heavier in δ^18^O and δ^2^H and plot farther off the LMWL compared to tidal channel samples, suggesting they have undergone more evaporation and are not simply depicting mixing between rainwater and seawater.

However, even though shrimp ponds are likely undergoing more evaporation, there is not a positive correlation with isotope values and salinity (SpC) (Additional file 1: Fig. A8). A positive correlation would be expected, because as water evaporates, conservative ions should stay in solution and become more concentrated. The lack of an observed positive correlation is likely a consequence of each shrimp pond having different starting salinities following irrigation from tidal channels, with concentration variations in the tidal channel irrigation source “masking” the relatively minor effects of evaporation on conservative salt ions. However, when looking at a shrimp pond and irrigating tidal channel directly adjacent to it, slight evaporative enrichment occurs with conservative elements, while several nonconservative elements are depleted in shrimp ponds (i.e., Mn and P; those less likely to desorb from saltwater cation exchange), possibly due to mineral precipitation or sorption onto sediment surfaces (Additional file 1: Fig. A9).

Evaporative models further illustrate the aforementioned point that although evaporation is likely occurring in the shrimp ponds more than the irrigating tidal channels, the amount of evaporation has little overall influence on major element concentrations in solution (Additional file 1: Fig. A5). Evaporation appears low in May (≤ 10%) and does not significantly affect element concentrations in shrimp ponds. This is likely a result of regular shrimp pond drainage of wastes and replenishment from new tidal channel water, which would reduce water residence time and the effects of evaporation. However, the evaporation models were based on a sample close to high tide and are thus minimum estimates of evaporation, although in the nearby Bangladesh Sundarbans, salinity changes between high and low tide in the dry season tidal channels are often around ~ 1ppt or less, with some tidal channels seeing practically no salinity change between tides at all [53, 54].

Trace elements of interest, such as As and Se, also do not show any clear trends with stable isotopes (Additional file 1: Fig. A10). Weak to moderate relationships are seen with all other elements as well (Additional file 1: Fig. A11). Essentially, evaporation is not great enough to overcome the large variance in starting compositions of the shrimp pond water, or the effects of nonconservative element behavior are larger than the effects of evaporation, effectively erasing any possible correlation between stable isotopes and nonconservative behaving elements.

DOC shows a strong positive relationship with stable isotopes (Fig. 7). As δ^18^O and δ^2^H isotopes become heavier in shrimp ponds, DOC increases as well, likely because these variables are affected by endogenous processes in shrimp ponds (e.g., biological processes or evaporation), while other geochemical parameters are more affected by exogenous processes (e.g., upstream rock weathering, seawater mixing). For example, as shrimp pond waters undergo more evaporation, they become more stagnant with lower flow conditions, algae-rich, and produce DOC through algal photosynthetic activity (e.g., [19]). Additionally, all shrimp ponds have similar DOC concentrations in their irrigation source (as seen with little variation in tidal channel DOC values in Fig. 7), which allows identification of the small changes caused by evaporation. DOC values do not show a relationship with isotopes in tidal channels because tidal channel isotopic values are predominantly influenced by rainwater-seawater mixing instead of evaporation.

Lastly, it must be noted that expanding isotopic analysis of shrimp pond waters to other months/seasons may be useful for helping determine geochemical relationships as well, particularly because when excluding isotopes, multidimensional scaling (MDS) shows July shrimp ponds are distinct from other samples (Additional file 1: Fig. A12).

### Multiple linear regression and predictors of As and Se

Nearly all our samples plotted similarly to seawater in a Piper diagram (Additional file 1: Fig. A13), and were thus collectively used in multiple linear regression to assess important predictor variables for As and Se, even though most elements showed large variability in concentration.

Multiple linear regression illustrates that Ni, P and V are important predictor variables for Se and/or As in most water samples (Fig. 8). Phosphorus is regarded as a “chemical analog” to As, and like As (as pentavalent arsenate) in oxidized aqueous environments, P is mainly in an oxyanion form (as PO_4_^3−^) [69]. However, in the main predictive model for As, P is positively correlated (Additional file 1: Table A3), which would suggest that even though both elements are likely negatively charged species in solution, their concentrations are not explained by competitive adsorption. Vanadium is likely predominantly an oxyanion as well given the pH and oxidizing conditions of the surface water samples, with the most common ion of V being H_2_VO_4_^−^ in natural waters [20]. Because V is positively correlated with As in the predictive model (Additional file 1: Table A3), it may also behave similarly in solution but not compete directly for sorption sites. However, Ni is most often a cation species with a valence of + 2 in natural waters [39]. When analyzing primary influencing variables for As (Cu, V, Ni and P), it is clear that only Ni is negatively correlated, illustrating that Ni may improve the model because of different chemical behaviors (i.e., sorption affinities) as a cation in solution (Additional file 1: Table A3).

Selenium also exists as oxyanions in oxidizing environments such as surface waters [73]. When looking at the predictive variables V, Ni, and DOC for Se,V has a negative coefficient in the model while Ni has a positive coefficient (Additional file 1: Table A5). This is opposite that of the main As model (Additional file 1: Table A3). Thus, for Se, even though V is likely an anion in solution and Ni a cation, the anion is associated with a decrease in Se in the model. This may be due to competitive sorption properties of Se and V on solid particle surfaces (particularly with selenite on Fe-oxyhydroxides or clay minerals), which would increase V in solution as Se sorbs. Ni may complex with aqueous species that behave similarly to Se but do not compete with sorption sites.

pH is important to include in modeling because of its known effects on speciation and mobility of Se and As (e.g., [69, 73]). However, models that include pH do not significantly improve As or Se prediction (Fig. 8), potentially due to relatively small variance in pH values (Table 1). Thus, pH does not likely have a large, statistically significant influence on either Se or As concentrations in these surface waters, although it is acknowledged that pH is important in speciation of these elements and thus their chemical characteristics in solution.

When adding isotopes to predictive modeling to determine whether evaporation/mixing may affect Se and As predictability, the adjusted R^2^ values for both Se and As increase to both 0.70 (p = 0.016) and 0.90 (p = 0.0041), respectively (Additional file 1: Tables A6, A7), but only 14 samples are included in the analysis, omitting all July shrimp ponds. The influence of isotopes must be taken with care, as clear bivariate relationships between Se, As and δ^18^O (and δ^2^H) are not apparent (Additional file 1: Fig. A10), and the inclusion of both isotopes (δ^18^O and δ^2^H) in the Se model (Additional file 1: Table A7) clearly violate the multiple linear regression assumption of no multicollinearity among predictor variables. However, future work investigating the predictive nature of stable isotopes for trace elements in surface water or groundwater modeling may be warranted because as proxies of mixing or evaporation, O and H isotopes may provide additional insight as to where certain elements originated from or if evaporation concentrated elements in solution.

Additionally, because organic matter such as DOC can affect trace elements in multiple ways, such as complexing with As species or competing for sorption sites (e.g., [41, 72]), it was another geochemical parameter worth examining. For Se, it was found that the predictor variables DOC, V, and Ni resulted in an adjusted R^2^ of 0.67 (p < 0.0001) (Additional file 1: Table A5). Thus, Se may also be affected by DOC, such as by surface water reoxidation of biogenic elemental Se (BioSe) that originated from microbes [73] in high DOC waters. More research in shrimp pond elemental cycling would help shed insight on this. Lastly, it is apparent that adding Cl as a representative saltwater ion to the variables DOC, V and Ni improves the predictive model (Additional file 1: Table A4), which may be indicative of slight seawater mixing or seasonality effects on aqueous Se. However, it is noted that Cl does have a strong positive correlation with V (Additional file 1: Fig. A1), and thus violates the assumption of no multicollinearity for multiple linear regression.

### Applicability of Se and As models

While these models do not identify the mechanisms that control As and Se concentrations in shrimp ponds and tidal channel waters, they are very useful in gauging what elements/geochemical parameters are most important in influencing As and Se concentrations in surface waters. This extends to upstream (non-headwaters) Ganges River samples [15], their Table S4), where Cu, Vi, and Ni result in a great multiple linear predictive model fit for As (adjusted R^2^ = 0.64, p = 7.2e-15) (Additional file 1: Table A8). Additionally, when examining tidal channel samples from Ayers et al. [8], predicting As with Cu, V, Ni, and P results in a great overall fit as well (adjusted R^2^ = 0 0.74, p = 0.024) (Additional file 1: Table A9). However, Se predictive modeling for tidal channels in Ayers et al. [8] is much poorer, with DOC, V, and Ni only resulting in an adjusted R^2^ of 0.24 (p = 0.17). Nevertheless, the general approach of multiple linear regression with several important predictor variables such as Ni and V or other geochemical analogues appears to be applicable to other surface water bodies, particularly rivers, and can be especially useful in better understanding and predicting the concentration of As in solution. Further research in quantifying Se in surface waters will help in understanding what controls/predicts its geochemical variability.

These models also provide a way to estimate As and Se concentrations in Bangladesh surface waters without direct measurements of As or Se. Model validation using other published datasets indicates that the model parameters obtained in this study do not produce model predictions that agree well with observed As and Se concentrations (i.e., Additional file 1: Fig. A14). However, the general approach used here can help improve predictions and measurements in other regions throughout the world, particularly when several geochemical parameters may be missing from samples within an area. For example, if a study has only analyzed As or Se in 15% of samples, but the remaining samples contain potentially important predictor variables such as Ni or V, a modeling solution can be applied to estimate As or Se in the remaining samples.

Although this study assumed linear relationships in predictive modeling, future work involving nonlinear predictive modeling techniques such as machine learning techniques (i.e., boosted regression trees) is warranted, as these techniques have been shown to outperform multiple linear regression predictive modeling in groundwater [49].

### Selenium and arsenic antagonistic relationship

Se is known to combat the toxic effects of As, and As is known to inhibit Se toxicity (e.g., [30, 67, 68]). Elevated Se and As can be taken up by biological organisms, and may eventually sorb onto soils/sediment. This is particularly relevant for As in the area, as many shrimp ponds are utilized for rice farming in the wet season [6], where flooded soils could remobilize sorbed As through reductive dissolution of Fe oxyhydroxides (e.g., [57]). However, even if Se becomes elevated in soils from tidal channel irrigation, the reducing conditions in flooded rice paddy soils could lead to immobile elemental selenium being formed, which is not bioavailable [73]. Thus, rice could potentially take up arsenic, but not selenium, with humans therefore lacking the detoxifying effects of selenium when ingesting the rice. Further work is needed to explore the cycling and antagonistic relationship between Se and As in Southwest Bangladesh, such as identifying if sediments in contact with Se and As-rich waters are elevated in both those elements, what fraction is bioavailable, and if Se concentrations in tidal channel waters in the peak monsoon season become heavily diluted. Although much research in Bangladesh has examined As in crops such as rice (e.g., [37, 71, 72]), crop/aquaculture Se should be more heavily researched in Southwest Bangladesh as well, particularly where Se water concentrations are high.

## Conclusion

Through examining surface water chemistry in Southwest Bangladesh in two different months over a wide spatial area, it was deduced that: (1) Monthly precipitation differences between May and July has a greater effect on tidal channel water composition compared to shrimp pond composition; (2) There is a large compositional difference between shrimp ponds and the tidal channels irrigating them in the early monsoon (July), but not in May; (3) Evaporation in shrimp ponds and endogenous pond effects in general have a relatively minimal impact on surface water trace element and major ion chemistry based on May pond data, although DOC has a strong positive correlation with δ^18^O and δ^2^H isotopes and thus evaporation; (4) Arsenic and selenium concentrations are elevated above WHO drinking water guidelines in the majority of surface water samples and are correlated with other trace elements in solution such as Ni and V; and (5) Predictive modeling of hazardous trace elements in surface waters may prove useful in future studies throughout the world when measurements of certain toxic elements cannot always be easily made.

## Supplementary Information

**Additional file 1** Supplementary tables and figures.

**Additional file 2.** A video of typical shrimp aquaculture pond irrigation with tidal channel water.

## Data Availability

All data is available within the manuscript and will be provided open access.
