# Supplementary material for: Influences on tidal channel and aquaculture shrimp pond water chemical composition in Southwest Bangladesh
Source: Geochem Trans. 2021 May 28;22:2. doi: 10.1186/s12932-021-00074-2 (PMC8164274; doi:10.1186/s12932-021-00074-2)
Supplement: Supplementary file 1 — Additional file 1 Supplementary tables and figures. [file 12932_2021_74_MOESM1_ESM.docx]

***Supplementary Information***

**Detailed Sampling Information:**

All acidified water samples (including blanks) except for rainwater underwent a 5:1 dilution with deionized water for ICP-MS analysis because of high salinity. The May 2019 water samples did not undergo dilution for the ICP-OES analysis, although July 2018 (excluding rainwater) and July 2019 water samples did undergo the 5:1 dilution. Non-diluted samples were still within the dynamic range of the ICP-OES. Figures/models contain data based on a 5:1 dilution, but reported data (Table 2) was updated for better precision based on the more specific 5.1:1 dilution. However, the 2% difference is less than the width of lines and points in the plots, and the dilution ratio change would not impact correlation matrices, nor evaporation and regression models.

Samples from 2019 were refrigerated after arrival to Vanderbilt University, while samples from 2018 were not, although they were analyzed within ~2 months for TOC/IC and the original ICP-OES run was completed within ~2 months as well. Ayers et al., 2017 did not see any significant changes in DIC or DOC over a period of 12-38 days, although nitrate did decrease, but is excluded in this study. Samples from 2019 were completely analyzed within ~2 months of sample collection, while ICP-MS and ICP-OES reanalysis for July 2018 final reported sample values was conducted ~15 months after sample collection because of initial concerns with potentially pushing the upper range of the ICP-OES quantification range for Na. However, the ICP-OES reanalysis concentrations were in excellent agreement for the major cations from the July 2018 samples’ original run, indicating evaporative concentration or precipitation of elements was unlikely, and volatilization of elements via atmospheric contact was also unlikely. It is noted though that absolute Na concentrations were consistently higher in the initial OES run.

The July 2018 tidal channel sample was not filtered for TOC or IC analyses. ICP-MS results are listed for As, because of better quantification with lower detection limits on the ICP-MS versus the ICP-OES.

Because shrimp pond KA-10 was not irrigated via tidal channels, it was excluded for all grouped shrimp pond comparisons. Although KA-3 was included because it was irrigated by a tidal channel, it may have an inaccurate SpC value (Table 1). Shrimp pond water samples in May were taken in close spatial proximity to shrimp pond sites in July (Fig. 2), so that spatial salinity gradients are unlikely to largely affect the interpretation of Fig. A3 or Fig. 4. It is noted that tidal channel sample Site-1 was more saline than any other samples (as seen through Na and Cl concentrations), but was likely under slightly different tidal influence and freshwater-seawater mixing dynamics than other samples (collected in the Kobodak cross channel), and was thus only included in isotope figures.

Additionally, the tidal channel sample (MD-TC-22) that was used in the evaporation models was sampled close to the time of high tide at the Pusur River mouth. This could mean the salinity values and isotopic values of the tidal channel sample were towards the upper range within daily fluctuating tidal cycles (higher tide means an isotope ratio enriched towards seawater and also greater seawater conservative ion concentrations) and thus provides an upper estimate of those measured inputs into the shrimp pond (and a minimum evaporation estimation).

**Multiple Linear Regression Modeling:**

Variables were selected for inclusion into multiple linear regression analyses initially based on selecting several elements showing moderate correlations with As or Se, such as V, Mn, DOC and Co (Fig. A1), or hypothesized geochemical relationships. For example, because Se shows distinct variance based on slight saltwater ion/salinity seasonality (Fig. 4; Fig. A3), Cl was included as a representative salt ion in modeling to determine whether it could serve as a predictive variable for Se. Rainwater was excluded in the modeling because it is not affected by local geochemical processes besides very minor atmospheric inputs, and thus most element concentrations are relatively negligible relative to seawater, tidal channel water, and groundwater (e.g., Ayers et al., 2017).

V and Cl are strongly positively correlated (Fig. A1), violating the assumption of no multicollinearity in multiple linear regression. When removing Cl from the predictive model of Cl, V, Ni, and DOC, the adjusted R^2^ becomes 0.67 (p <0.0001) with each variable (V, Ni and DOC) having a p-value <0.01 (Table A5), which is greater than the adjusted R^2^ (0.64, n = 30, p <0.0001) for Co, Ba and DOC even though these variables appear to be the best predictive variables for a 3 variable model when 25 samples are considered (Fig. 8). Only four samples with missing values for any of the As predictive variables (n = 26) and only one sample with missing values for any of the Se predictive variables (n = 29) were omitted from the aforementioned models (Tables A3, A4, and A5).

Using the variable subset of Se, P, V, Ni, δ^18^O, and pH resulted in an adjusted R^2^ of 0.70 for As (p = 0.016) (Table A6), and selecting the variables Al, Co, P, Ba, Ni, δ^18^O, δ^2^H and pH resulted in an adjusted R^2^ of 0.90 for Se (p = 0.0041) (Table A7). However, when including isotopes, 16 sample observations must be omitted due to missing variables, making a sample size of n = 14. Furthermore, having both δ^18^O and δ^2^H as predictor variables clearly violates the assumption of no multicollinearity for multiple linear regression. When omitting δ^18^O from the Se model, the adjusted R^2^ drops to 0.45 (p = 0.14) and omitting δ^2^H reduces the R^2^ to 0.42 (p = 0.16).

**Tables and Figures**

**Table A1:** Concentrations of elements in seawater compiled from the literature: Pilson, 1998 (^a^), Sarma et al., 2012 – MN Transect (^b^), Ogawa and Tanoue, 2003 – Upper range of Indian Ocean values (^c^), Srichandan et al., 2016 – Upper limit off NW Bay of Bengal (^d^), Mason, 2013 (^e^), and Wright and Colling, 1995 (^f^). For simplicity, all elements are listed at total concentrations, although major elements (^a^) and DIC (^b^) in seawater are derived from their predominant ion concentrations in mg/kg but written here as mg/L because the density conversion is negligible (~1.02) for an already generic estimation.

|  | Seawater Concentration (mg/L) |  | Seawater Concentration (mg/L) |
| --- | --- | --- | --- |
| **Cl^a^** | **19353** | **Ba^f^** | **0.02** |
| **Na^a^** | **10781** | **Al^e^** | **0.00054** |
| **SO4^a^** | **2712** | **P^f^** | **0.06** |
| **Mg^a^** | **1284** | **As^e^** | **0.0017** |
| **S^f^** | **905** | **Se^e^** | **0.00013** |
| **K^a^** | **399** | **V^e^** | **0.0015** |
| **Ca^a^** | **411.9** | **Mn^d^** | **0.00734** |
| **DIC^b^** | **19.1** | **Zn^e^** | **0.00033** |
| **DOC^c^** | **0.84** | **Cu^e^** | **0.00025** |
| **Sr^a^** | **7.94** | **Ni^e^** | **0.00047** |
| **B^a^** | **25.7** | **Cr^e^** | **0.00021** |
| **Si^f^** | **2** | **Co^e^** | **0.0000012** |

**Table A2:** Geochemist’s Workbench React program script for a surface water evaporation model, modeling evaporation of tidal channel input water while in the irrigated shrimp pond.

data = thermo.tdat verify

conductivity = conductivity-USGS.dat

temperature = 25 C

H2O = 1 free kg

Al+++ = 93.7 ug/l

V+++ = 18.1 ug/l as V

Cr+++ = 4.4 ug/l as Cr

Mn++ = 4.1 ug/l as Mn

Co++ = .36 ug/l as Co

Ni++ = 5.6 ug/l as Ni

Cu+ = 5.9 ug/l as Cu

Zn++ = 4.1 ug/l as Zn

As(OH)4- = 42.6 ug/l as As

SeO3-- = 11.7 ug/l as Se

B(OH)3 = 2.5 mg/l as B

Ba++ = .25 mg/l as Ba

Ca++ = 198.6 mg/l as Ca

K+ = 278.2 mg/l as K

Mg++ = 567 mg/l as Mg

Na+ = 5405 mg/l as Na

HPO4-- = .054 mg/l as P

SO4-- = 1337 mg/l

SiO2(aq) = 2.12 mg/l as Si

Sr++ = 3.65 mg/l as Sr

Cl- = 8869 mg/l

Br- = 44.86 mg/l

pH = 7.67

swap e- for O2(aq)

Eh = 267 mV

HCO3- = 112.5 mg/l

balance off

react -100 g/l of H2O

**Table A3:** Multiple linear regression output for selected primary predictor variables based on multiple linear subset regression for As.

Call:

lm(formula = As ~ Cu + P + V + Ni, data = data)

Residuals:

Min 1Q Median 3Q Max

-0.020069 -0.008693 -0.002284 0.005545 0.048094

Coefficients:

Estimate Std. Error t value Pr(>|t|)

(Intercept) -0.006741 0.012124 -0.556 0.5841

Cu 1.718763 0.687983 2.498 0.0209 *

P 0.077547 0.051316 1.511 0.1456

V 2.041054 0.575027 3.549 0.0019 **

Ni -1.863999 0.753860 -2.473 0.0220 *

---

Signif. codes: 0 ‘***’ 0.001 ‘**’ 0.01 ‘*’ 0.05 ‘.’ 0.1 ‘ ’ 1

Residual standard error: 0.01448 on 21 degrees of freedom

(4 observations deleted due to missingness)

Multiple R-squared: 0.4839, Adjusted R-squared: 0.3855

F-statistic: 4.922 on 4 and 21 DF, p-value: 0.005861

**Table A4**: Multiple linear regression output for selected primary predictor variables based on multiple linear subset regression for Se.

Call:

lm(formula = Se ~ V + Ni + DOC + Cl, data = data)

Residuals:

Min 1Q Median 3Q Max

-0.032661 -0.019022 -0.002155 0.013897 0.040920

Coefficients:

Estimate Std. Error t value Pr(>|t|)

(Intercept) 1.416e-02 1.317e-02 1.075 0.292912

V -5.476e+00 1.327e+00 -4.127 0.000382 ***

Ni 3.178e+00 9.707e-01 3.274 0.003212 **

DOC 5.713e-03 1.232e-03 4.638 0.000104 ***

Cl 7.832e-06 2.706e-06 2.895 0.007962 **

---

Signif. codes: 0 ‘***’ 0.001 ‘**’ 0.01 ‘*’ 0.05 ‘.’ 0.1 ‘ ’ 1

Residual standard error: 0.02211 on 24 degrees of freedom

(1 observation deleted due to missingness)

Multiple R-squared: 0.7802, Adjusted R-squared: 0.7436

F-statistic: 21.3 on 4 and 24 DF, p-value: 1.317e-07

**Table A5**: Multiple linear regression output for selected primary predictor variables based on multiple linear subset regression for Se.

Call:

lm(formula = Se ~ V + Ni + DOC, data = data)

Residuals:

Min 1Q Median 3Q Max

-0.039108 -0.019917 -0.003914 0.013867 0.059160

Coefficients:

Estimate Std. Error t value Pr(>|t|)

(Intercept) 0.028225 0.013932 2.026 0.05358 .

V -2.183840 0.777885 -2.807 0.00954 **

Ni 4.782861 0.906714 5.275 1.83e-05 ***

DOC 0.003774 0.001176 3.208 0.00364 **

---

Signif. codes: 0 ‘***’ 0.001 ‘**’ 0.01 ‘*’ 0.05 ‘.’ 0.1 ‘ ’ 1

Residual standard error: 0.02516 on 25 degrees of freedom

(1 observation deleted due to missingness)

Multiple R-squared: 0.7035, Adjusted R-squared: 0.6679

F-statistic: 19.77 on 3 and 25 DF, p-value: 8.798e-07

**Table A6:** Multiple linear regression output including an isotope predictor variable for As.

Call:

lm(formula = As ~ Se + P + V + Ni + d18O + pH, data = data)

Residuals:

Min 1Q Median 3Q Max

-0.0059425 -0.0008563 0.0000130 0.0007339 0.0047557

Coefficients:

Estimate Std. Error t value Pr(>|t|)

(Intercept) 0.133264 0.043684 3.051 0.01857 *

Se -0.281884 0.123210 -2.288 0.05599 .

P 0.099795 0.022593 4.417 0.00309 **

V 2.113018 0.601300 3.514 0.00981 **

Ni 2.184351 0.863182 2.531 0.03920 *

d18O 0.004651 0.001765 2.635 0.03367 *

pH -0.017973 0.005586 -3.218 0.01470 *

---

Signif. codes: 0 ‘***’ 0.001 ‘**’ 0.01 ‘*’ 0.05 ‘.’ 0.1 ‘ ’ 1

Residual standard error: 0.003919 on 7 degrees of freedom

(16 observations deleted due to missingness)

Multiple R-squared: 0.8387, Adjusted R-squared: 0.7005

F-statistic: 6.068 on 6 and 7 DF, p-value: 0.01596

**Table A7:** Multiple linear regression output including isotope predictor variables for Se.

Call:

lm(formula = Se ~ Al + Co + P + Ba + Ni + d18O + d2H + pH, data = data)

Residuals:

13 14 15 16 17 18 19

3.579e-03 1.681e-03 4.400e-03 6.696e-05 -3.326e-03 -5.290e-04 -2.544e-03

20 21 22 23 24 25 26

-4.228e-03 2.542e-04 -8.720e-04 -5.446e-04 9.332e-04 -1.351e-03 2.481e-03

Coefficients:

Estimate Std. Error t value Pr(>|t|)

(Intercept) 6.138e-01 8.430e-02 7.281 0.000764 ***

Al -9.558e-01 2.329e-01 -4.104 0.009316 **

Co -4.591e+02 7.981e+01 -5.753 0.002227 **

P -8.565e-01 1.430e-01 -5.991 0.001858 **

Ba 1.629e-01 6.939e-02 2.347 0.065798 .

Ni 3.139e+00 8.493e-01 3.697 0.014048 *

d18O -2.140e-01 4.105e-02 -5.213 0.003429 **

d2H 3.773e-02 7.052e-03 5.350 0.003064 **

pH -3.119e-02 5.868e-03 -5.315 0.003152 **

---

Signif. codes: 0 ‘***’ 0.001 ‘**’ 0.01 ‘*’ 0.05 ‘.’ 0.1 ‘ ’ 1

Residual standard error: 0.004016 on 5 degrees of freedom

(16 observations deleted due to missingness)

Multiple R-squared: 0.9604, Adjusted R-squared: 0.8969

F-statistic: 15.14 on 8 and 5 DF, p-value: 0.004144

**Table A8**: Multiple linear regression output for important predictor variables for As from data in Table S4 in Boral et al. (2020), from the upper Ganges.

Call:

lm(formula = As ~ Cu + V + Ni, data = PredictD)

Residuals:

Min 1Q Median 3Q Max

-11.8380 -2.8581 -0.0012 2.5799 14.2559

Coefficients:

Estimate Std. Error t value Pr(>|t|)

(Intercept) -1.65994 1.30592 -1.271 0.20830

Cu 4.75747 0.52821 9.007 5.5e-13 ***

V -0.27914 0.09889 -2.823 0.00634 **

Ni -0.81865 0.50824 -1.611 0.11216

---

Signif. codes: 0 ‘***’ 0.001 ‘**’ 0.01 ‘*’ 0.05 ‘.’ 0.1 ‘ ’ 1

Residual standard error: 4.771 on 64 degrees of freedom

Multiple R-squared: 0.6569, Adjusted R-squared: 0.6408

F-statistic: 40.84 on 3 and 64 DF, p-value: 7.19e-15

**Table A9:** Multiple linear regression output for important predictor variables for As from tidal channel surface water data (including a duplicate sample) in Ayers et al. (2020), from lower Ganges tidal tributaries.

Call:

lm(formula = As ~ Cu + V + Ni + P, data = Ayers_TC)

Residuals:

1 3 4 5 6 7 10

-1.645e-04 -1.880e-04 3.680e-04 8.622e-06 -3.094e-04 -1.952e-04 -5.321e-04

11 12 13

-6.370e-04 4.793e-04 1.170e-03

Coefficients:

Estimate Std. Error t value Pr(>|t|)

(Intercept) 0.0044004 0.0003822 11.513 8.67e-05 ***

Cu -0.2447471 0.0814662 -3.004 0.02995 *

V 0.4528870 0.1766426 2.564 0.05041 .

Ni -1.0276585 0.6569294 -1.564 0.17851

P -0.1493359 0.0316770 -4.714 0.00527 **

---

Signif. codes: 0 ‘***’ 0.001 ‘**’ 0.01 ‘*’ 0.05 ‘.’ 0.1 ‘ ’ 1

Residual standard error: 0.0007238 on 5 degrees of freedom

(5 observations deleted due to missingness)

Multiple R-squared: 0.8576, Adjusted R-squared: 0.7436

F-statistic: 7.526 on 4 and 5 DF, p-value: 0.02407


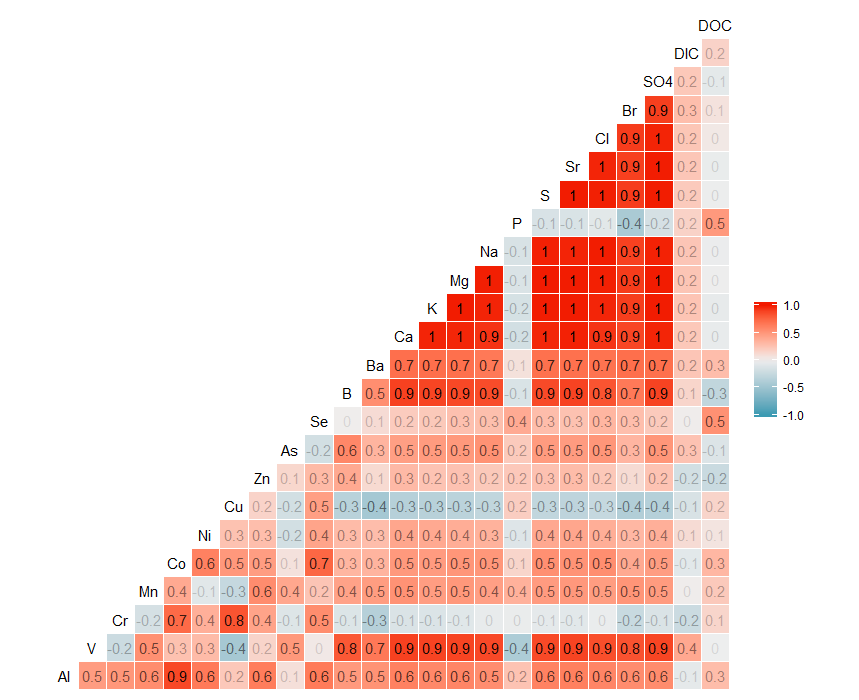
**Figure A1:** Pearson correlation coefficient matrix between log10 transformed dissolved element concentrations in surface water samples, excluding rainwater (Si omitted because of several negative values, and pH, ORP (Eh) and SpC omitted because of missing values).


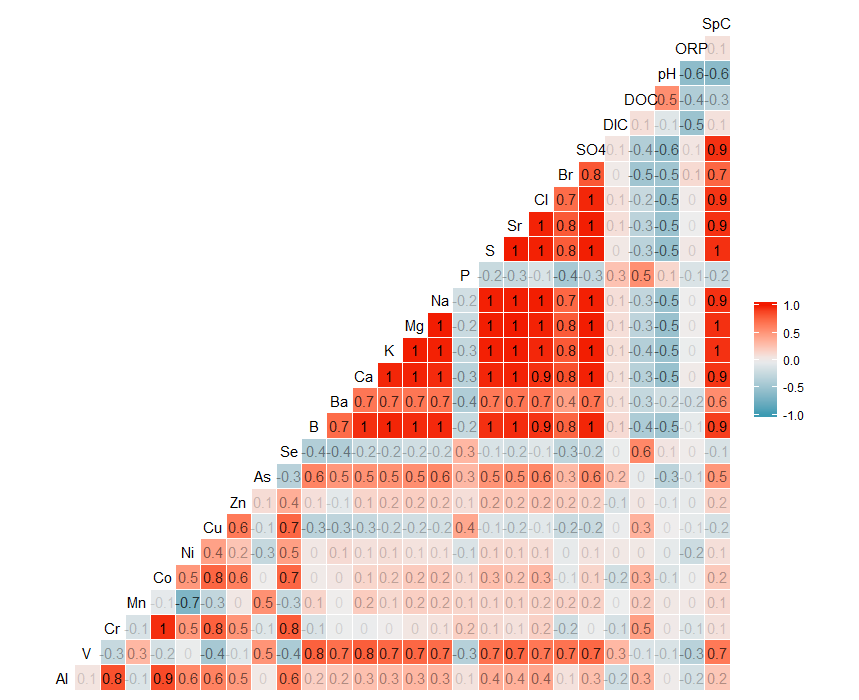


**Figure A2:** Pearson correlation coefficient matrix between log10 transformed dissolved element concentrations in shrimp pond samples (Si omitted because of several negative values). For the correlation matrix, ORP (relative to Ag/AgCl redox couple) is interchangeable with Eh even though Eh values relative to the standard hydrogen electrode (SHE) are +187 mV compared to ORP values.


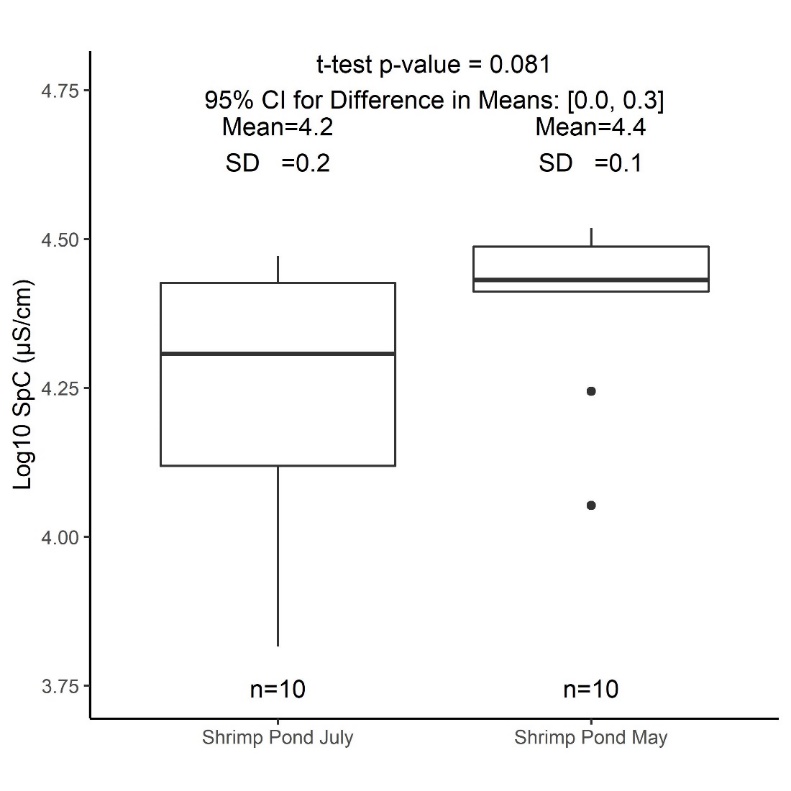


**Figure A3:** Comparative box plot illustrating the slight seasonality seen between July and May shrimp ponds irrigated with tidal channels via log transformed SpC data.

**Figure A4**: Spider diagram showing relative enrichment or depletion of elements in tidal channel water when normalized to average seawater concentrations (Table A1; values above 0 are enriched relative to seawater, values <0 are relatively depleted).

**Figure A5:** Geochemist’s Workbench evaporation model output, depicting specific conductivity (SpC) in solution during evaporation of a tidal channel sample towards the composition of the irrigated shrimp pond sample. The model’s initial SpC values calculated from the analysis are slightly different from the field-measured value, likely because of discrepancies between the Geochemist’s Workbench SpC calculation and greater uncertainty of field measurements at high salinity.


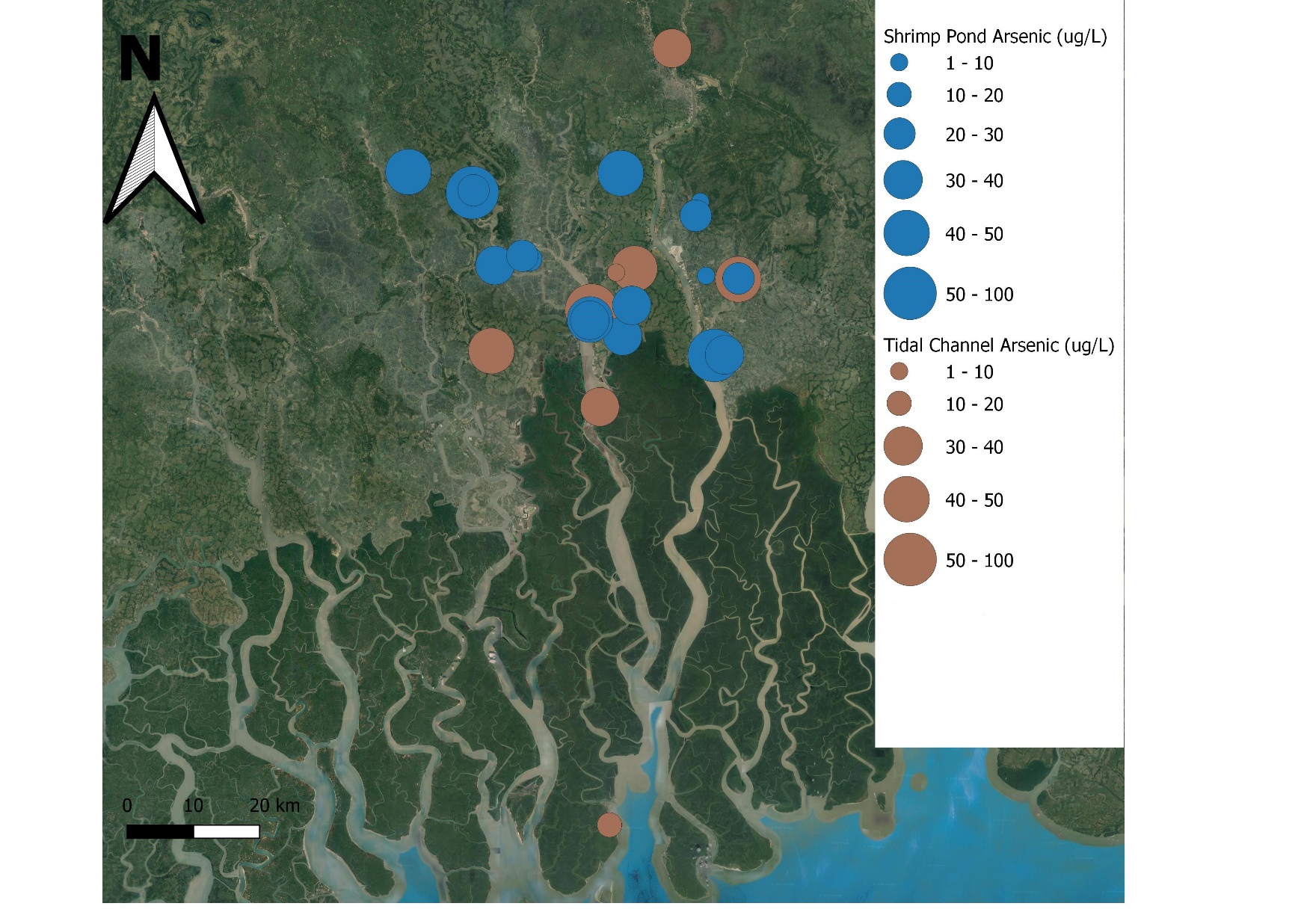


**Figure A6**: Arsenic concentrations in shrimp ponds and tidal channels from May and July 2018-2019. Large spatial heterogeneity is seen, with most concentrations over the WHO guideline value of 10 μg.L^-1^.


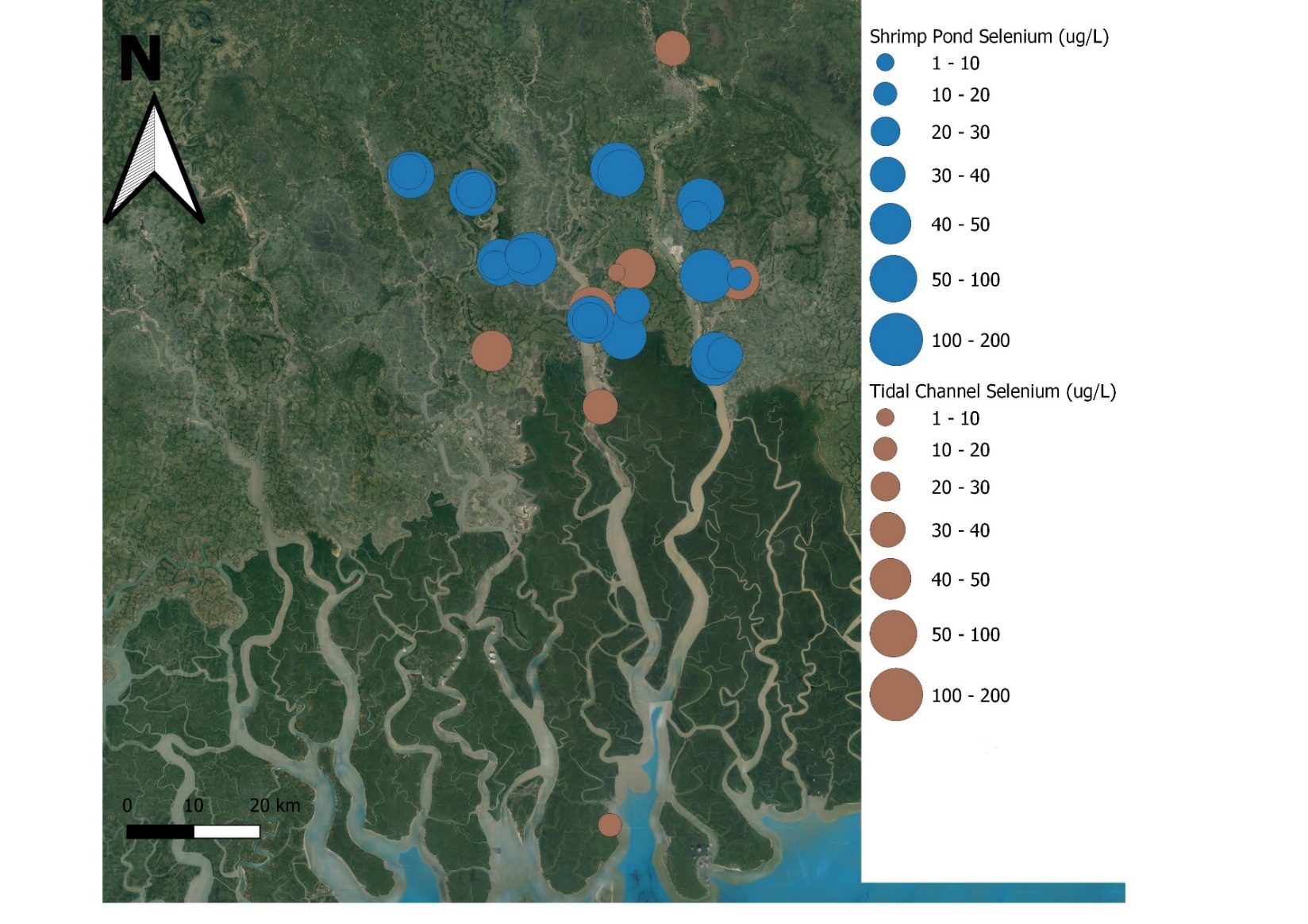


**Figure A7**: Selenium concentrations in shrimp ponds and tidal channels from May and July 2018-2019. Large spatial heterogeneity is seen, with many samples over the EPA MCL of 50 μg.L^-1^.

**Figure A8:** Bivariate plot between SpC and δ^18^O in May shrimp ponds.

**Figure A9:** Bar chart plot of ratio between concentration in shrimp pond and a tidal channel directly adjacent to it as an irrigation source. All values >1 (red line) indicate higher shrimp pond values than tidal channel values.


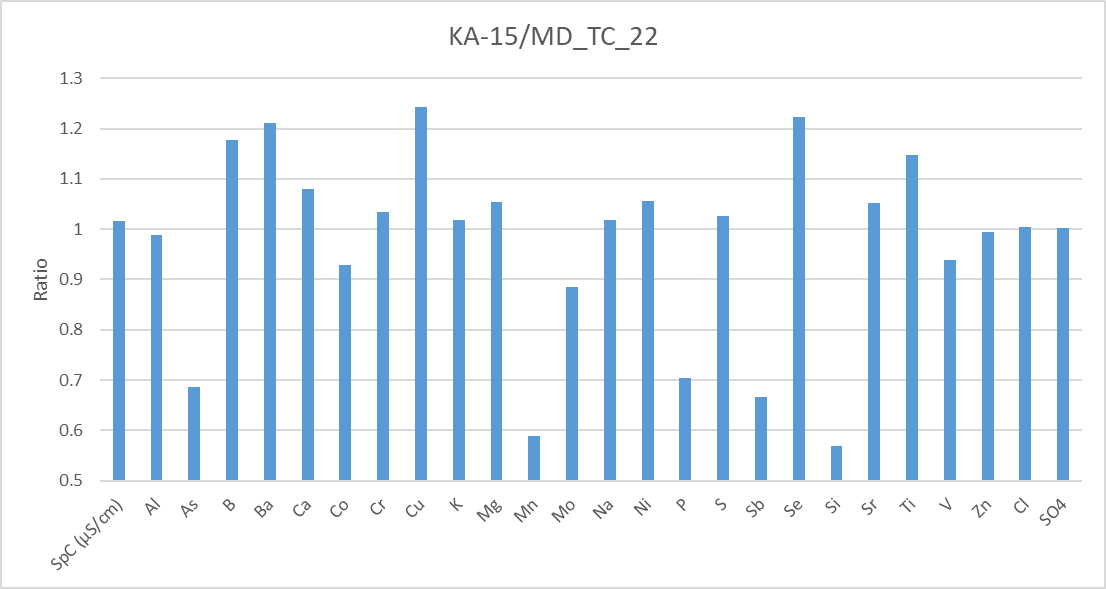


**Figure A10:** Bivariate plot of As and Se (μg.L^-1^) with δ^18^O, showing little to no relationship.


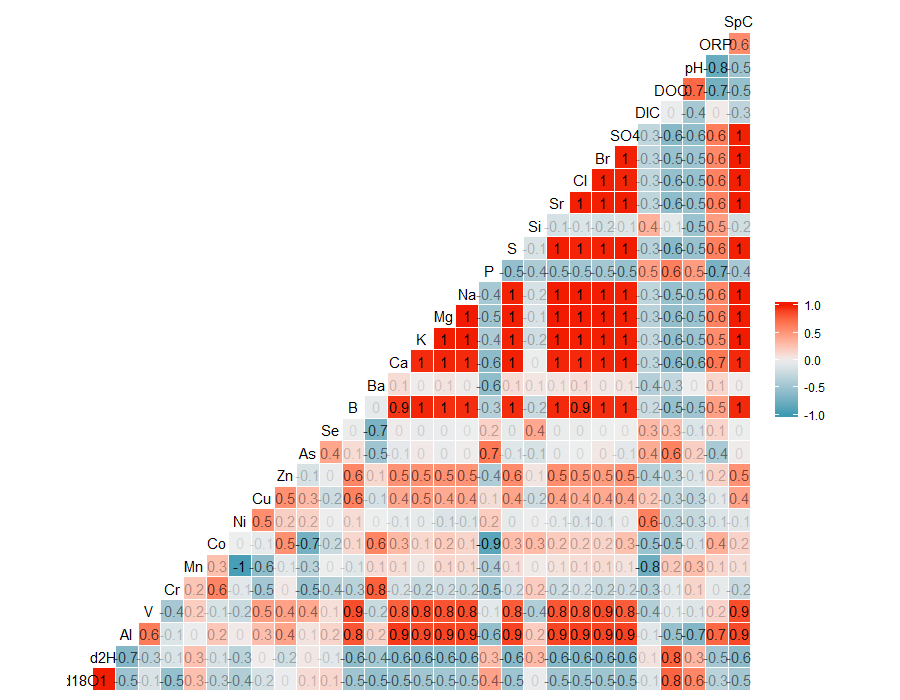


**Figure A11:** Log10 transformed Pearson correlation coefficient matrix between δ^18^O and δ^2^H, elements and other geochemical parameters in May shrimp pond water samples. For the correlation matrix, ORP (relative to Ag/AgCl redox couple) is interchangeable with Eh even though Eh values relative to the standard hydrogen electrode (SHE) are +187 mV compared to ORP values.


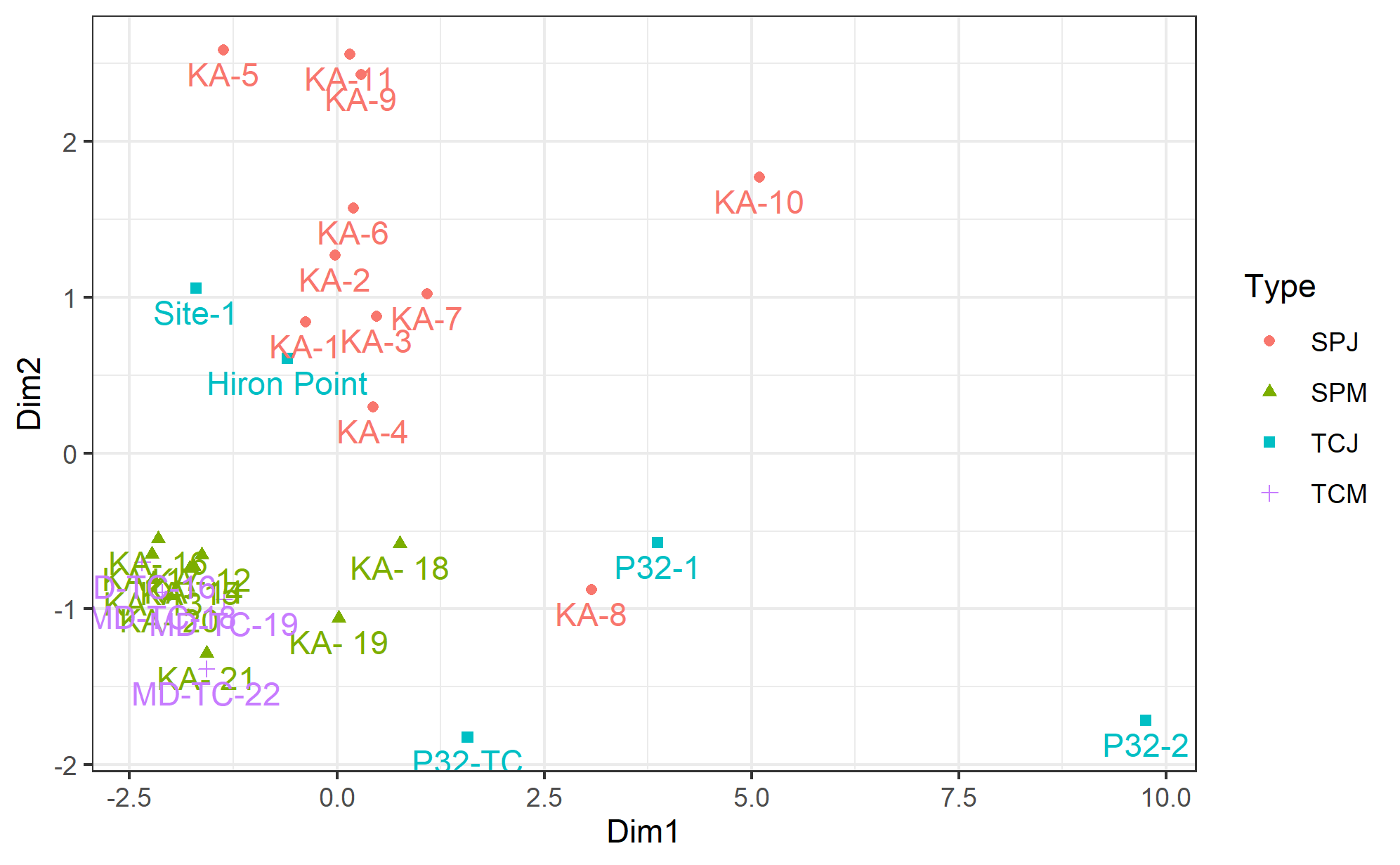


**Figure A12:** Multidimensional scaling (MDS) plot by Aitchison distance (using the classical method) of July shrimp ponds (SPJ), May shrimp ponds (SPM), July tidal channels (TCJ), and May tidal channels (TCM) for all geochemical variables except stable isotopes, with missing data replaced via the “mice” package (van Buuren, 2020) and MDS completed via the “provenance” package (Vermeesch, 2020). In addition to illustrating the compositional difference between July and May samples, MDS shows the similarity in May tidal channel and shrimp pond composition, and the disconnect among July shrimp ponds and tidal channels. MDS is useful because it plots samples based on multiple compositional variables in abstract Cartesian space, where samples that are more similar plot closer together.


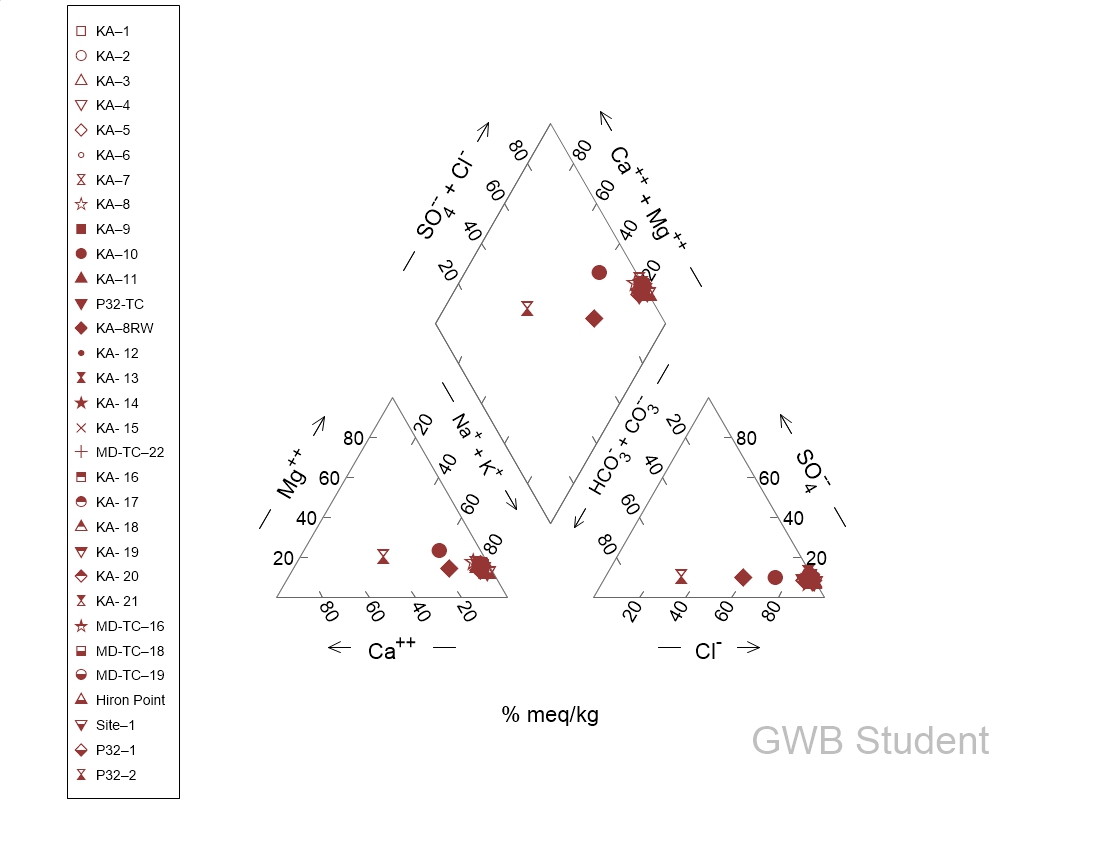


Typical Seawater

**Figure A13:** Piper diagram created in The Geochemist’s Workbench, illustrating how most water samples plot within typical seawater composition.


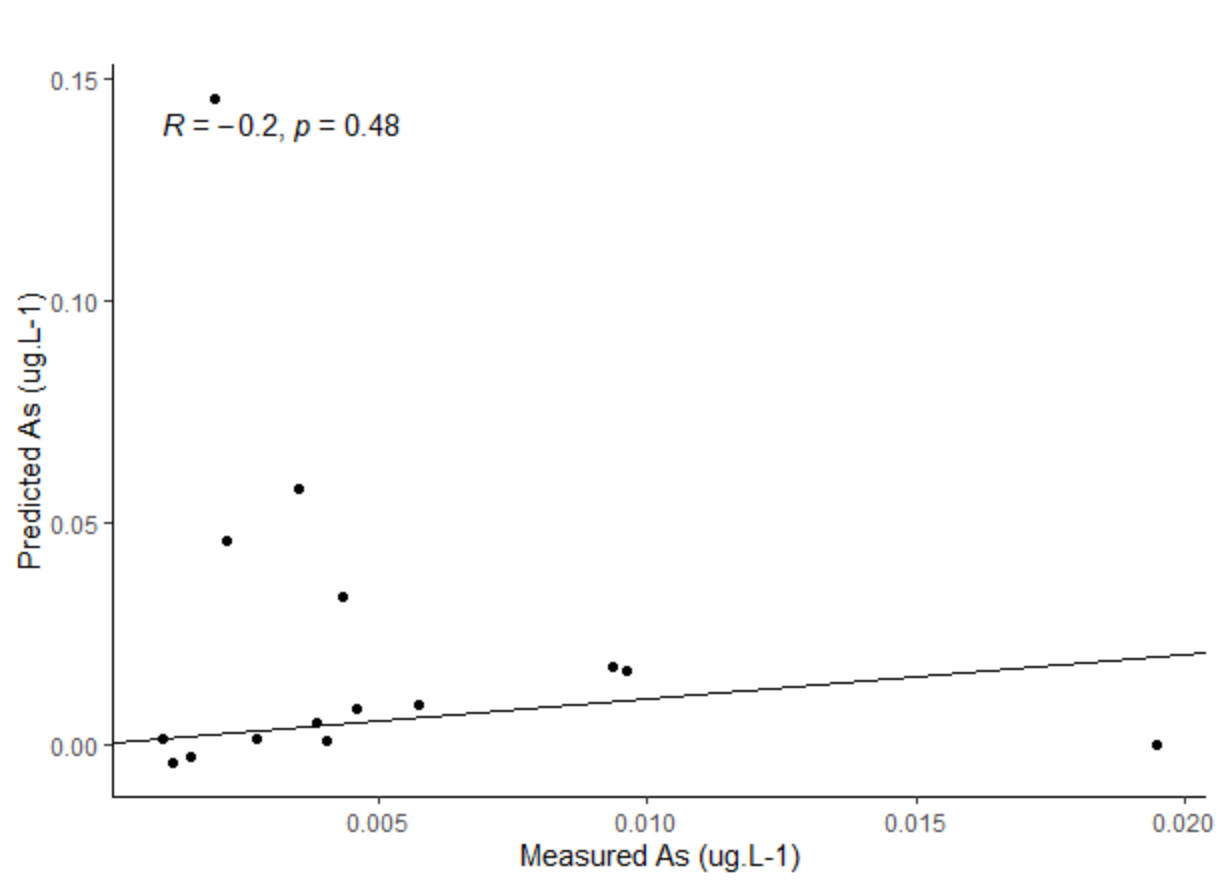

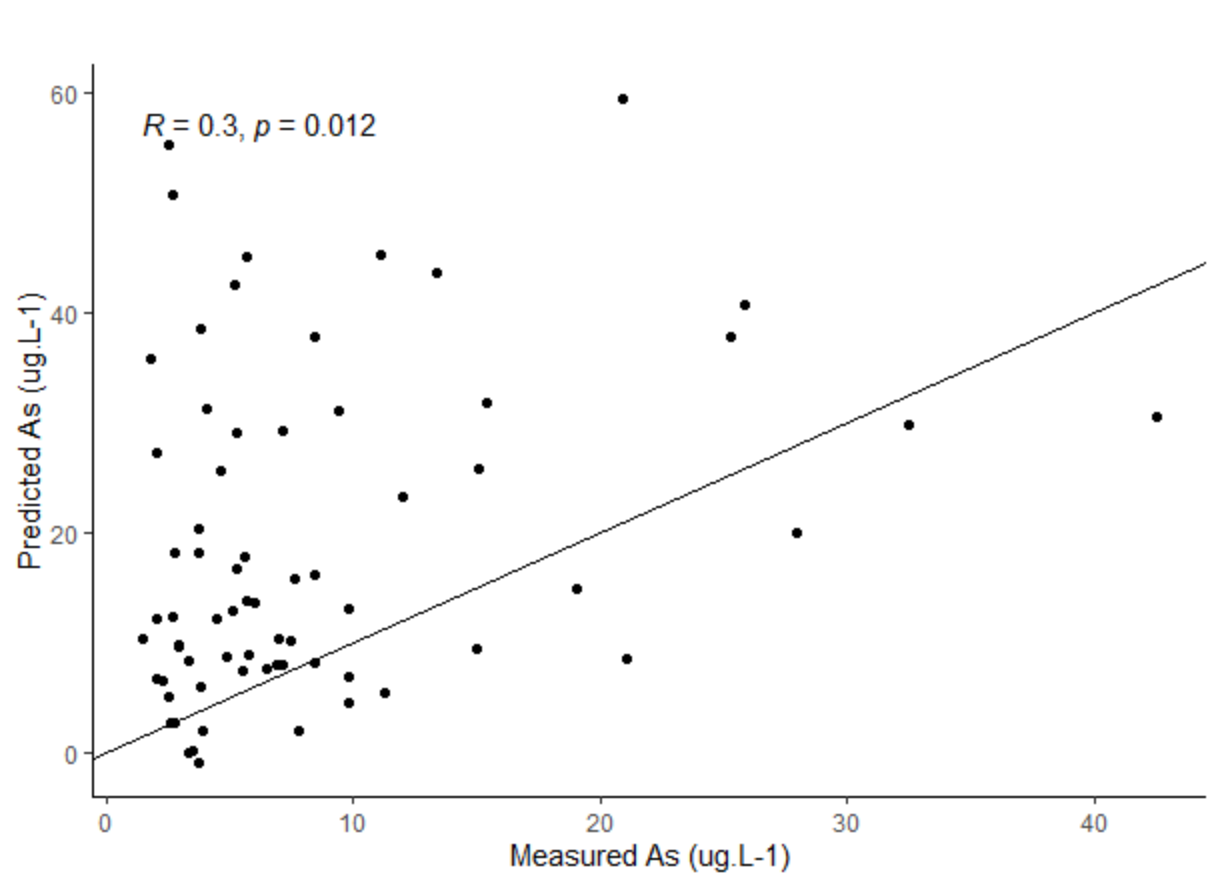


A

B

**Figure A14:** Predicted versus measured As in surface water samples in (A) the Ganges River (Boral et al., 2020) and (B) Ganges tidal channel tributaries (Ayers et al., 2020), with the solid black line representing a 1:1 ratio. A Pearson correlation coefficient is also provided for each plot.

**References**

Ayers, J.C., George, G., Fry, D., Benneyworth, L., Wilson, C., Auerbach, L., Roy, K., Karim, M.R., Akter, F., Goodbred, S., 2017. Salinization and arsenic contamination of surface water in southwest Bangladesh. Geochem. Trans. 18, 4. <https://doi.org/10.1186/s12932-017-0042-3>

Ayers, J. C., Patton, B., & Dietrich, M., 2020. Preliminary Evidence of Transport‐Limited Chemical Weathering and Element Immobility in the Ganges Tidal Delta Plain of Bangladesh. *Geochemistry, Geophysics, Geosystems*, *21*(8), e2020GC009029. <https://doi.org/10.1029/2020GC009029>

Boral, S., Sen, I.S., Tripathi, A., Sharma, B., Dhar, S., 2020. Tracking dissolved trace and heavy metals in the Ganga River from source to sink: A baseline to judge future changes. Geochemistry, Geophys. Geosystems 1–22. <https://doi.org/10.1029/2020GC009203>

van Buuren, S., 2020. Multivariate Imputation by Chained Equations. <https://cran.r-project.org/web/packages/mice/mice.pdf>

Mason, R. P., 2013. Trace metals in aquatic systems. John Wiley & Sons.

Ogawa, H., Tanoue, E., 2003. Dissolved Organic Matter in Freshwaters. J. Oceanogr. 59, 129–147. <https://doi.org/10.1023/A:1025528919771>

Wright, J., Colling, A., 1995. Seawater: Its Composition, Properties and Behaviour. Elsevier. <https://linkinghub.elsevier.com/retrieve/pii/C20130102085>

Pilson, M. E. Q., 1998. Major constituents of seawater. An Introduction to the Chemistry of the Sea, 58-65.

Sarma, V.V.S.S., Krishna, M.S., Rao, V.D., Viswanadham, R., Kumar, N.A., Kumari, T.R., Gawade, L., Ghatkar, S., Tari, A., 2012. Sources and sinks of CO 2 in the west coast of Bay of Bengal. Tellus, Ser. B Chem. Phys. Meteorol. 64. <https://doi.org/10.3402/tellusb.v64i0.10961>

Srichandan, S., Panigrahy, R.C., Baliarsingh, S.K., Rao B., S., Pati, P., Sahu, B.K., Sahu, K.C., 2016. Distribution of trace metals in surface seawater and zooplankton of the Bay of Bengal, off Rushikulya estuary, East Coast of India. Mar. Pollut. Bull. 111, 468–475. <https://doi.org/10.1016/j.marpolbul.2016.06.099>

Vermeesch, P., 2020. provenance: Statistical Toolbox for Sedimentary Provenance Analysis. <https://cran.r-project.org/package=provenance>
